# Electrochemical β‐Selective Hydrocarboxylation of Styrene Using CO_2_ and Water

**DOI:** 10.1002/advs.201900137

**Published:** 2019-12-17

**Authors:** Younghye Kim, Gyeong Do Park, Mani Balamurugan, Jiwon Seo, Byoung Koun Min, Ki Tae Nam

**Affiliations:** ^1^ Department of Materials Science and Engineering Seoul National University Seoul 151‐744 Republic of Korea; ^2^ Clean Energy Research Center Korea Institute of Science and Technology 5 Hwarang‐ro 14‐gil, Seongbuk‐gu Seoul 02792 Republic of Korea; ^3^ Department of Chemistry School of Physics and Chemistry Gwangju Institute of Science and Technology Gwangju 61005 Republic of Korea

**Keywords:** carbon fixation, carboxylation, CO_2_ reduction, electrochemistry

## Abstract

The carboxylation of hydrocarbons using CO_2_ as a one‐carbon building block is an attractive route for the synthesis of carboxylic acids and their derivatives. Until now, chemical carboxylation catalyzed by organometallic nucleophiles and reductants has been generally adopted particularly for the precise selectivity control of carboxylation sites. As another approach, electrochemical carboxylation has been attempted but these carboxylation reactions are limited to only a few pathways. In the case of styrene, dicarboxylation at the α‐ and β‐positions is mostly observed with electrochemical carboxylation while site‐selective hydrocarboxylations are hardly achieved. In this study, electrochemical β‐selective hydrocarboxylation of styrene using CO_2_ and water is developed, in which the site selectivity can be precisely controlled between β‐hydrocarboxylation and dicarboxylation without the aid of homogeneous catalysts. In this platform, water is used as proton source in the β‐hydrocarboxylation of styrene where its addition results in significant enhancement of the selectivity toward β‐hydrocarboxylation. This work provides insights into new strategies for site‐selectivity‐controllable carboxylation with CO_2_ using an electrochemical platform.

## Graphical abstract

The carboxylation of styrene on a heterogeneously catalyzed electrochemical platform is newly demonstrated, where CO_2_ and water are used as carbon and proton sources, respectively. The site‐selectivity of carboxylation can be precisely controlled between dicarboxylation and β‐selective hydrocarboxylation solely by providing optimal proton source in the absence of homogeneous catalysts.

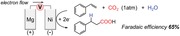

## 1 Introduction

Carbon dioxide (CO_2_) fixation in natural photosynthesis (e.g., RuBisCO) occurs by carboxylation of unsaturated carbon bond in enediolate intermediates.^1^ Recently, a similar mechanism of CO_2_ insertion at the unsaturated carbon bond has been adopted in the synthesis of carboxylic acids employing alkynes,^2^ α‐olefins^3^ and internal alkenes as substrates.^4^ These methods enabled CO_2_ to be harnessed as a renewable one‐carbon building block; however, the valorization of CO_2_ is still challenging because the gas is thermodynamically and kinetically stable.^5^ Consequently, numerous advances in chemical carboxylation using CO_2_ have relied on highly reactive organometallic nucleophiles to facilitate the reaction.^5^, ^6^ Recent reports have demonstrated that the elaborate design of organometallic nucleophiles is the primary requisite for modulation of site‐selectivity and extension of substrates in carboxylation.^6^, ^7^ Recently, Martin and co‐workers developed an elegant protocol for site‐selectivity tunable carboxylation via nickel hydride or nickelalactone formation for the extensive scope of unsaturated hydrocarbons, such as styrenes, alkenes and alkynes.^8^

As an alternative approach, heterogeneously catalyzed electrochemical carboxylation has gained increasing attention. This reactions are mainly driven by reductive electrical potential on a cathode electrode.^9^ Therefore, the reduction reaction takes place in the absence of a reducing agent and the reducing power can be easily controlled by the applied potentials. Among the unsaturated hydrocarbon feedstocks, this study focused on the electrochemical carboxylation of styrene as a representative model.

In the carboxylation of styrene using CO_2_, hydrocarboxylation of the α‐ or β‐position and dicarboxylation at both positions are feasible. Vianello and co‐workers first pioneered the electrochemical dicarboxylation of styrene to form 2‐phenylsuccinic acid (1).^10^ Since then, several succeeding works on the electrochemical carboxylation of styrene have reported dicarboxylation as a primary reaction both in the presence^11^ and absence^12^ of homogeneous catalysts. These studies proposed the electrochemical formation of β‐carboxylate radical anions as a key intermediate, followed by additional CO_2_ insertion to the benzylic position.[qv: 12a,c] It has been hypothesized that the electrochemical carboxylation of styrene is mostly carried out by the dicarboxylation pathway, whereas addition of water as protic agent can change reaction pathway to β‐hydrocarboxylation.[qv: 11b,12d] Although these study showed that the selectivity of carboxylation can be controlled in electrochemical platform, the mechanistic study on the reaction selectivity is rarely conducted.

In contrast, chemical carboxylation of styrene has been actively studied, ranging from dicarboxylation to hydrocarboxylation either with Markovnikov or anti‐Markovnikov regioselectivity. Notably, pioneering works have progressed to modulate site‐selectivity by developing novel chemical protocols,^13^ metal catalysts,^14^ and catalyst ligands.[qv: 14a,b,15] Most of the works using metal catalysts reported α‐hydrocarboxylation as a major reaction due to the preference for forming stable η^3^ benzylic metal species.^14^, ^16^ This process facilitates Markovnikov hydrocarboxylation where the proton of the metal hydride first binds to the β‐position and then CO_2_ later binds to the benzylic position. In the case of β‐hydrocarboxylation, CO_2_ insertion at the β‐position occurs prior to protonation which is the reverse order to that for α‐hydrocarboxylation as noted recently by the Jamison group[qv: 13b] and the König group.[qv: 15a] Both works achieved high selectivity for the β‐position by activating CO_2_ in the initial process of carboxylation using photocatalysts.

**Figure 1:**
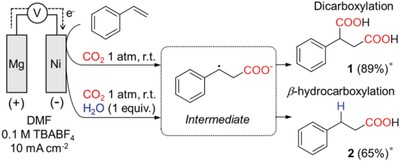
Electrochemical carboxylation of styrene with CO_2_. *Faradaic efficiency.

Inspired by these predictive selectivities in chemical carboxylation, we envisioned that electrochemical carboxylation would be extended to site‐selective hydrocarboxylation by controlling the protonation process. Because β‐carboxylate is the key intermediate during electrical reduction, we thought that intentional protonation might suppress successive CO_2_ insertion to form 1 (**Figure** **1**, top) and result in β‐hydrocarboxylation to form hydrocinnamic acid (2) (Figure 1, bottom). As previous works demonstrated,[qv: 11b,12d] water can be used as a clean and abundant proton source β‐hydrocarboxylation. Therefore, we explored the effect on the yield and selectivity of carobxylation products in the presence of water. Gaseous products from CO_2_ reduction and water reduction were also measured to precisely investigate the overall electrochemical reaction pathway. Solely by adding water, the selectivity toward β‐hydrocarboxylation enhanced from 3% (in the absence of water) to 96% and exhibited the maximum faradaic yield of 65%. Also, the effect of other proton sources and working electrodes were investigated and mechanistic study was conducted by isotope labeling experiments. Basis on these results, we proposed a new reaction pathway for electrochemical carboxylation of styrene that can selectively carry out toward dicarboxylation and β‐hydrocarboxylation.

## 2 Results and Discussion

**Figure 2:**
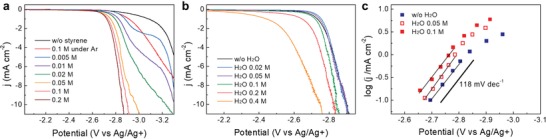
Voltammetric measurements of styrene. The measurements were conducted on a Ni electrode in CO_2_‐saturated *N*,*N*‐dimethylformamide and tetrabutylammonium tetrafluoroborate (0.1 m) electrolyte at room temperature and 1 atm CO_2_. a) Cathodic linear sweep voltammetry data of different concentrations of styrene (0 to 0.2 m) at 20 mV s^−1^. b) Cathodic linear sweep voltammetry data of 0.1 m styrene with various concentrations of H_2_O (0 to 0.4 m) at 20 mV s^−1^. c) Tafel plots from voltammetric measurements of 0.1 m styrene in the presence of 0, 0.05, and 0.1 m H_2_O. Current densities are calculated from the partial current densities of carboxylation of styrene.

We first performed the electrochemical carboxylation of styrene using CO_2_ without any proton source. Three electrode systems with a Ni cathode, Mg anode and Ag/Ag^+^ (0.01 m)/ tetrabutylammonium tetrafluoroborate (TBABF_4_, 0.1 m) reference electrode were used in *N,N*‐dimethylformamide (DMF) and TBABF_4_ (0.1 m) electrolyte. To investigate voltammetric behavior of styrene, linear sweep voltammetry scans were recorded at a 20 mV s^−1^ scan rate with IR compensation (**Figure** **2**a). The onset reduction potential is defined as the potential at the current density of 0.1 mA cm^−2^. As shown in the enlarged scans (Figure S1a, Supporting Information), onset potential of styrene (0.1 m) reduction reaction in Ar‐saturated electrolyte is −2.71 V (vs Ag/Ag^+^), which is more negative by 0.05 V compared to that of CO_2_ reduction reaction (CO_2_‐saturated, −2.66 V vs Ag/Ag^+^). Then, various concentration of styrene up to 0.2 m were added to the CO_2_‐saturated electrolyte. In the case of adding 5 × 10^−3^ m styrene, the onset potential was same as that of CO_2_‐saturated electrolyte without styrene. As adding more styrene, the onset potential shifted positively and maintained the value at −2.50 V, while the current density gradually enhanced. The increase of current density reached saturation in the case of styrene concentration above 0.1 m because of the diffusion limitation of styrene. Therefore, the concentration of styrene was fixed to 0.1 m in the following analysis.

The effect of protons on styrene carboxylation was investigated by adding various concentrations of water. First, linear sweep voltammetry scans were recorded by enhancing the water concentrations up to 0.4 m at a 20 mV s^−1^ scan rate with IR compensation (Figure 2b). The onset potential was observed at identical potential until the water concentration increased to 0.05 m and shifted to positive direction at higher water concentrations above 0.1 m. Also, as shown in the enlarged cyclic voltammetry scan with 0.1 m H_2_O (Figure S1b, Supporting Information), new reduction peak which has the current density of ≈0.2 mA cm^−2^ was observed from the applied potential at −1.53 V (vs Ag/Ag^+^). These results indicate the occurrence of a new reduction reaction in the presence of water which turned out to be a hydrogen evolution reaction (HER), as will be discussed later. To determine the rate‐determining step (RDS), Tafel slopes were measured for the cases of 0, 0.05, and 0.1 m water, where a negligible shift of the onset potentials was observed (Figure 2c). For the Tafel slope analysis, partial current density of carboxylation reaction was used by considering the yield of products. The measured slope value was between 113 and 120 mV dec^−1^, which suggests that one electron is involved in the RDS.^17^ As a result, we thought that the formation of CO_2_^•−^ or [Styrene]^•−^ could correspond to the RDS.

For the electrochemical electrolysis, 20 C of charge was passed under constant negative potential. As shown in Figure 2a, the reduction current highly depends on the concentration of the styrene, especially at low concentrations. Here, because the passed charge was not sufficient to consume the initial styrene, the current density of 10 mA cm^−2^ was stably maintained during electrolysis for ≈2000 s, as shown in Figure S2 (Supporting Information). Thus, the product yield could be measured in stable electrochemical reaction condition. The carboxylate products were acidified with HCl (2 m), extracted with ether and isolated after solvent distillation. The final carboxylic acids were characterized using proton nuclear magnetic resonance spectroscopy (^1^H NMR, 400 MHz, in DMSO‐*d*6). The ^1^H NMR spectra clearly showed the change in peak intensity of 1 and 2 by adding water (Figure S3, Supporting Information). In the spectra, the peaks of residual styrene, DMSO‐*d*6, DMF and water were observed but no other peaks of by products such as polymerized or reduced styrenes are detected. In addition, α‐hydrocarboxylated products were not detected both from NMR and gas chromatography‐mass spectrometry (GC‐MS) analysis. Along with the carboxylic acids in the electrolyte, the gas products from the headspace of the reactor, such as hydrogen, carbon monoxide and methane, were analyzed by gas chromatography (GC).

**Figure 3:**
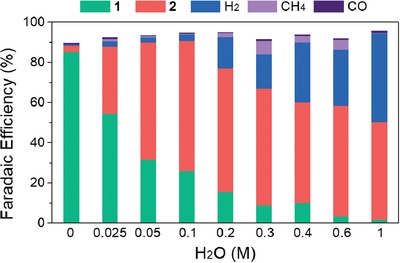
Faradaic efficiency (%) of products from electrochemical carboxylation of styrene. Electrolysis was conducted with 0.1 m styrene and various amounts of H_2_O on a Ni electrode in CO_2_‐saturated *N*,*N*‐dimethylformamide and tetrabutylammonium tetrafluoroborate (0.1 m) electrolyte with *j* = 10 mA cm^−2^, charge passed = 20 C, room temperature and *P*_CO2_ = 1 atm. The products in the electrolyte were acidified with HCl (2 m) and extracted with ether for ^1^H NMR detection (1 and 2). The gas products (H_2_, CO, and CH_4_) in the reactor headspace were detected by gas chromatography.

Based on the ^1^H NMR and GC results, the yields of each product from the styrene carboxylation were obtained based on faradaic efficiency (FE) in the presence of different amounts of water (**Figure** **3** and Table S1: Supporting Information). Among acid products, the selectivity of 2 to 1 dramatically increased from 3% to 96% as the water concentration increased from 0 m (Table S1, entry 1, Supporting Information) to 1 m (Table S1, entry 11, Supporting Information). This result clearly shows a change in the carboxylation pathway from dicarboxylation to β‐hydrocarboxylation. However, in terms of product yield, the maximum FE of 2 was obtained at 0.1 m water which is 1 equivalent relative to styrene (65%, Table S1, entry 5, Supporting Information) and gradually decreased to 47% when water was added up to 1 m (Table S1, entry 11, Supporting Information). This decline of yield toward β‐hydrocarboxylation can be explained by the increasing gas production. In particular, FE of hydrogen significantly increased from 3.2% to 15% as the water concentration increased from 0.1 to 0.2 m (Table S1, entries 5 and 7, Supporting Information) and even increased up to 44% at 1 m water (Table S1, entry 11, Supporting Information). Among the CO_2_ reduction reactions, the production of CH_4_ gradually increased, exhibiting a maximum FE of 6.7% in 0.3 m water (Table S1, entry 8, Supporting Information). The result indicates that excess water above 0.1 m (1 equivalent relative to styrene) in the electrolyte is used mostly in the HER and partially in CH_4_ production. This increase in gas production also corresponds to the positive shift of the onset potential in voltammetry scans at water concentrations above 0.1 m (Figure 2b). Consequently, it is clearly evident that the amount of water present in the reaction system is crucial in controlling both the selectivity and reactivity in the carboxylation of styrene.

In addition, the effect of water acidity on the product selectivity was investigated by using diluted HCl. In detail, the equivalent amount of 2 m HCl in terms of protons was used instead of 0.05 and 0.1 m neutral water, which was expected to enhance the free proton concentrations to pH 2.74 and 2.44, respectively (Table S1, entries 4 and 6, Supporting Information). The selectivity of 2 was slightly enhanced in both cases using HCl, which were comparable to the cases that used a twofold amount of neutral water.

The effect of electrode catalyst was investigated by using Ti and Pt cathode instead of Ni cathode. Ni, Ti, and Pt are in the metal group that prefers HER than CO_2_ reduction reaction.^18^ To use CO_2_ as a carbon source in the carboxylation, CO_2_ molecule should not be reduced directly into hydrocarbon products such as CO and CH_4_. Thus, we expected that these cathodes can increase the carboxylation efficiency while suppressing CO_2_ reduction reaction. For the electrochemical analysis, linear sweep voltammetry scans were recorded with 0, 0.05, 0.1, and 0.4 m water at a 20 mV s^−1^ scan rate in CO_2_‐saturated DMF and TBABF_4_ (0.1 m) electrolyte under an atmosphere of CO_2_ (Figure S4a,b, Supporting Information). By adding water, the onset potentials shifted positively and new currents from HER were observed as also observed in the case of Ni cathode. Especially, Pt electrode exhibited the largest current density of HER (0.5 mA cm^−2^ with 0.1 m H_2_O) among the electrodes used in this study. The acids and gas products from the styrene carboxylation using Ti and Pt electrode were also detected under the same electrolysis condition which was used in the case of Ni electrode in Figure 3. The calculated FE values of products using Ti and Pt electrode are shown in Figure S4c,d (Supporting Information). In both cases, the trends in the change of products under 0 to 0.4 m water was analogous to the case of Ni electrode, where the yield of 1 decreased while that of 2 and HER increased by adding water. However, compared to a Ni electrode, both cases showed lower yield of 2 in the presence of water. In the case of Pt electrode, as also observed in the voltammetry scans, it exhibited the highest activity for HER where the FE of hydrogen reached to 38% and 61% at 0.1 and 0.4 m water while the maximum yield of 2 was only 29% at 0.4 m water. On a Ti electrode, the HER were observed by FE of 18% and 40% at 0.1 and 0.4 m water and maximum yield of 2 was 44% at 0.1 m water.

Various protic solvents with increasing p*K*_a_ value were also used instead of water to explore the effect of proton sources on the selectivity toward 2 (**Table** **1**). Initially, we assumed that acidity of protic solvents can be a major determinant of carboxylation selectivity and employed alcohols and carboxylic acids with various p*K*_a_ values as protic solvents. However, the trend of the product selectivity showed no clear correlation to the p*K*_a_ value and it was confirmed that water was the most favorable proton source for the β‐hydrocarboxylation of styrene. This result reflects the complicated involvements of various determinants in heterogeneously catalyzed system,which includes reactant accessibility to electrode, binding affinity on the catalyst and interaction between proton source and substrates. Therefore, the verification of exact mechanism on proton donating process in this reaction condition requires further investigations.

**Table 1:**
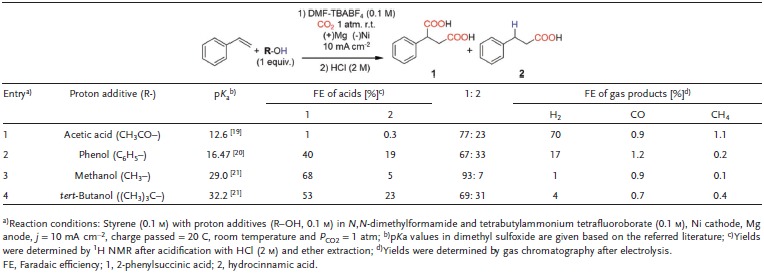
Effect of proton source type on the electrochemical carboxylation of styrene

The influence of current density was also investigated by applying various overpotentials (η) during the electrolysis (Table S2, Supporting Information). To evaluate β‐hydrocarboxylation activity, the turnover frequency (TOF) was calculated (see the Supporting information). When the current density was below 5 mA cm^−2^, the HER was mainly observed because of the insufficient overpotentials for carboxylation to be favored dominantly over the HER. When overpotential was increased to 320 mV, a dramatic enhancement of the acid products was observed, yielding 91% along with a high TOF of 10 s^−1^ (Table S2, entry 3, Supporting Information). The TOF further reached 38 s^−1^ at a 760 mV overpotential (Table S2, entry 5, Supporting Information) along with the negligible change in the selectivity between 1 and 2.

**Figure 4:**
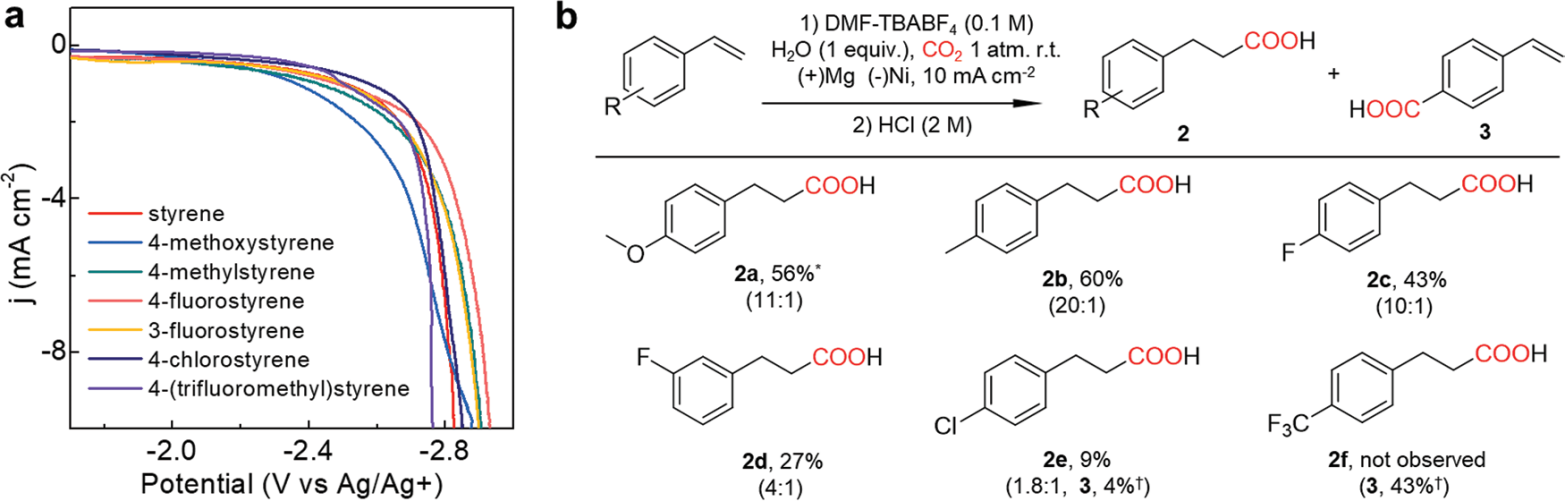
Scope of styrene derivatives for electrochemical β‐hydrocarboxylation. a) Cathodic linear sweep voltammetry data of 0.1 m styrene derivatives with 0.1 m H_2_O at 50 mV s^−1^. b) Yield of products. The ratio between β‐hydrocarboxylated and dicarboxylated product are shown in brackets. The electrochemical carboxylation of styrene derivatives (0.1 m) was conducted on Ni electrode in CO_2_ saturated *N*,*N*‐dimethylformamide and tetrabutylammonium tetrafluoroborate (0.1 m) electrolyte with *j* = 10 mA cm^−2^, charge passed = 20 C, room temperature and *P*_CO2_ = 1 atm in the presence of 0.1 m H_2_O. *Faradaic efficiency determined by ^1^H NMR after acidification with HCl (2 m) and ether extraction. †Isolated yields based on the reactant styrenes detected by ^1^H NMR after acidification with HCl (2 m) and ether extraction.

After optimizing the reaction condition for the β‐hydrocarboxylation of styrene, the substrate generality of this electrochemical protocol was investigated (**Figure** **4**). The condition that exhibited maximum yield of 2 using 1 equivalent water was utilized for the reactions. First, cathodic linear sweep voltammetry was measured at 50 mV s^−1^ to evaluate reduction activities. Compared to styrene, styrene derivatives bearing electron‐donating substituents such as methoxy group showed positively shifted onset potential while electron‐neutral and electron‐withdrawing styrenes possessed comparable or positively shifted onset potentials. The electrochemical carboxylation reactions were conducted with 0.1 m of each styrene derivatives and 0.1 m water on a Ni electrode in CO_2_‐saturated DMF and TBABF_4_ (0.1 m) electrolyte under 10 mA cm^−2^ current density. As a result, the β‐hydrocarboxylation reaction tolerated electron‐donating (2a) and electron‐neutral (2b) styrenes in good yields and fluorine‐substituted styrenes (2c and 2d) with moderate yields. In the cases of 2e and 2f, 4‐vinylbenzoic acid (3) was synthesized as a result of substitution of chlorine or trifluoromethyl group with CO_2_. Especially, when chlorine‐substituted styrene was used, bare styrene was observed after the electrochemical reaction and 1 and 2 were observed by FE with 16% and 59%, which clearly shows the cleavage of chlorine. However, no other styrene derivatives underwent the cleavage to styrene. Moreover, for trifluoromethyl‐substituted styrene, no products from the cleavage of fluorine‐carbon bond was observed under our detection condition using NMR and GC‐MS analysis. To further investigate the reduction of carbon‐chlorine bond, current densities during the carboxylation of chlorine‐substituted styrene were changed to 1, 10, and 20 mA cm^−2^. As a result, neither carboxylation toward 2e nor reduction of carbon‐chlorine bond were occurred under 1 mA cm^−2^ current density, and negligible change in product selectivity was observed for the cases of 10 and 20 mA cm^−2^ current density. The behavior of electron‐withdrawing styrenes exhibits similar trend with previous electrochemical carboxylation of styrene without proton source[qv: 12c] which also shows the cleavage (or reduction) of carbon‐chlorine bond instead of the reduction of vinyl group.

**Figure 5:**
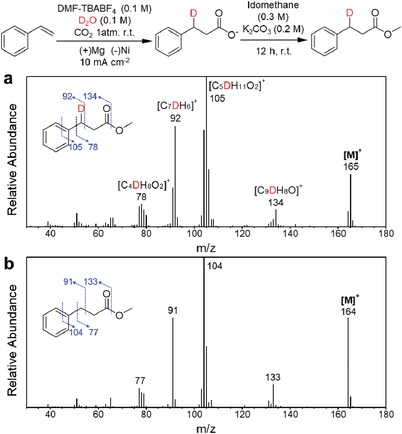
Gas chromatography‐mass spectrometry (GC‐MS) results from deuterium labeling experiments using D_2_O. The carboxylate products from the electrochemical carboxylation of styrene (0.1 m) using D_2_O or H_2_O (0.1 m) as the proton source were methylated with iodomethane (0.3 m) with K_2_CO_3_ (0.2 m) and extracted with ether for GC‐MS analysis. The mass spectra of a) deuterium labeled methyl‐3‐phenylpropanoate obtained from the case using D_2_O and b) methyl‐3‐phenylpropanoate from the case using H_2_O are shown. Electrochemical carboxylation of styrene (0.1 m) was conducted on a Ni electrode in CO_2_‐saturated *N*,*N*‐dimethylformamide and tetrabutylammonium tetrafluoroborate (0.1 m) electrolyte with *j* = 10 mA cm^−2^, charge passed = 20 C, room temperature and *P*_CO2_ = 1 atm. The molecular ion peaks ([*M* ^+^]) of deuterium labeled methyl‐3‐phenylpropanoate and methyl‐3‐phenylpropanoate were observed at *m*/*z* 165 and 164, respectively.

To verify whether the proton used in β‐hydrocarboxylation comes from water, a deuterium labeling experiment was conducted by fully replacing water with deuterium oxide (D_2_O). In the ^1^H NMR spectra, D‐labeled 2 was observed with 80% deuterium incorporation at the benzylic carbon site (see the Supporting Information). Moreover, carboxylic acid products were methylated and observed in GC‐MS (see the Supporting Information). In the mass spectrum, the ion peak (*M* ^+^) of D‐labeled methyl‐3‐phenylpropanoate showed a mass shift of 1 from *m*/*z* 164 to 165, which corresponds to labeling with one deuterium atom (**Figure** **5**). Also, four molecular fragments of D‐labeled methyl‐3‐phenylpropanoate (*m*/*z* 78, 92, 105, and 134) showed a mass shift of 1. The only carbon position that these four fragments commonly contain is the benzylic carbon which indicates that deuterium labeling has been carried out at this site. Therefore, basis on the result from both ^1^H NMR and GC‐MS, we clearly verified that the protonation occurs at a benzylic position by using a proton of water.

After verifying the proton participation in this β‐hydrocarboxylation, electrokinetic study was investigated by using D_2_O as a proton source. In particular, the influence of the protonation step on the overall electrochemical carboxylation of styrene was explored in terms of kinetics. First we recorded cyclic voltammetry scans of the electrochemical carboxylation of styrene (0.1 m) with 0.025 and 0.1 m D_2_O or H_2_O. At these proton source concentrations, the selectivity of carboxylation over gas production was above 94%, which could avoid the contribution of other unwanted proton‐involved reactions such as the HER. As shown in Figure S5 (Supporting Information), no difference was observed in the voltammetric curves between the cases using H_2_O and D_2_O. This result implies that a protonation step is not involved in the RDS of this electrochemical carboxylation. In addition, we calculated the yield of acid products from the carboxylation and found out that total FE of acids (1 + 2) were only slightly changed from 88% to 85% (entry 2, **Table** **2**) and 91% to 86% (entry 4, Table 2). This trend, the independency on H/D exchange, corresponds to the result of cyclic voltammetry scan. However, at the same time, the selectivity of β‐hydrocarboxylation toward dicarboxylation significantly decreased when D_2_O was used. As a result, the FE of 2 were reduced in the D‐labeled cases, from 33% to 20% (entry 2, Table 2) and 65% to 44% (entry 4, Table 2), respectively. Along with other results, this data shows that the protonation step play a major role in tuning the selectivity between carboxylation pathways between dicarboxylation and β‐hydrocarboxylation.

**Table 2:**
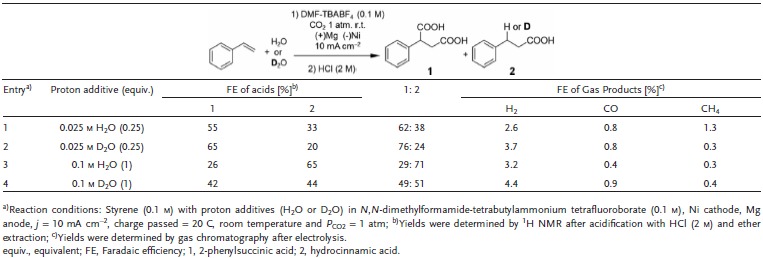
Effect of deuterium labeled proton source on the product yield of the electrochemical carboxylation of styrene

Finally, radical inhibitors such as (2,2,6,6‐Tetramethylpiperidin‐1‐yl)oxyl (TEMPO) and 5,5‐dimethyl‐pyrroline N‐oxide (DMPO) were added to the reaction solution to investigate the role of radical intermediates during the electrochemical carboxylation of styrene (Figure S6, Supporting Information). In the case of using TEMPO and DMPO, the FE of 1 decreased from 26% to 8% and 13% respectively which shows the suppression of dicarboxylation in the presence of the inhibitors. Whereas, the FE of 2 showed comparable value (68%, with TEMPO) or even enhanced (86%, with DMPO). These changes in the electrochemical carboxylation pathway in the presence of radical inhibitors indicate that radical intermediate play an essential role in this electrochemical reaction. Also, we added benzoquinone (BQ), which can act as a proton scavenger, and observed enhancement of FE toward 1 (49%) while that of 2 decreased to 27%. This shows that the supply of proton is necessary to facilitate the hydrocarboxylation pathway.

**Figure 6:**
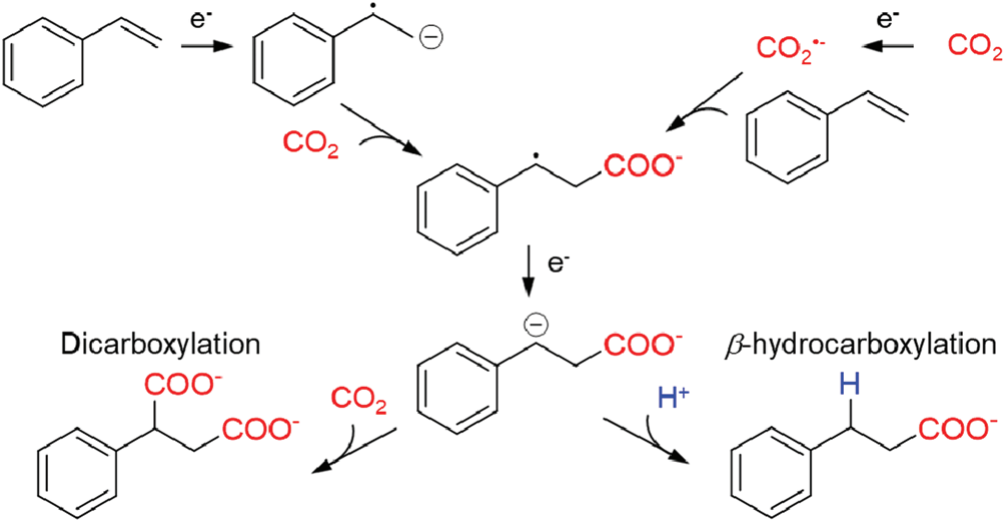
Proposed mechanism of the electrochemical carboxylation of styrene.

Based on our collective results, we proposed a reaction pathway for electrochemical β‐hydrocarboxylation of styrene in the presence of small amount of water (**Figure** **6**). First, styrene and CO_2_ form a β‐carboxylate radical intermediate by accepting one electron. The absence of α‐hydrocarboxylated products in study supports the formation of β‐carboxylated species as an intermediate at the initial stage of the reaction. This electrical reduction step is the RDS as confirmed by the Tafel analysis and electrokinetic study. From the result that the reduction current has gradually increased by adding reactants, it can be presumed that styrene and CO_2_ simultaneously undergo reduction reaction at the applied potential. In terms of onset potential, CO_2_ reduction reaction showed more positive onset potential (−2.66 V vs Ag/Ag^+^) compared to the styrene reduction reaction (−2.71 V vs Ag/Ag^+^) by 50 mV. However, under the reaction condition used here which applies at least 320 mV overpotential, it is still ambiguous to decide the dominant pathway between these two reactions. Because the reduction potential of benzylic radicals are positioned at more positive range than the onset potential (between −1.82 to −0.71 V vs SCE, in acetonitrile),^22^ the β‐carboxylate radical intermediate could be reduced to β‐carboxylate anion. This would avoid unwanted reaction among styrene radicals and result in the absence of polymerized or reduced styrenes during the reaction. Next, protonation takes place at a benzylic position by using a proton, as proved by deuterium labeling analysis, and this protonation leads the reaction to produce 2 via the β‐hydrocarboxylation pathway. In the study, diluted HCl solution showed higher selectivity toward β‐hydrocarboxylation than pure water which implies the proton donating ability affects the protonation efficiency. Whereas, using different protic solvent instead of water showed no clear relation between the acidity of solvents and the product selectivity. The dicarboxylation pathway can be explained by the additional incorporation of CO_2_ into the benzylic position of the β‐carboxylate anion intermediate. Consequently, the competition between the protonation and CO_2_ incorporation on the benzylic position determines the final selectivity of this electrochemical carboxylation.

## 3 Conclusion

In summary, a new electrochemical carboxylation of styrene with CO_2_ was demonstrated where the site selectivity could be controlled between dicarboxylation and β‐hydrocarboxylation by inducing protonation of styrene using water. With 10 equivalents of water relative to styrene, the selectivity of β‐hydrocarboxylation toward dicarboxylation reached up to 96%. However, because excess water facilitated the HER and methane production instead of carboxylation, the highest FE of β‐hydrocarboxylation was observed when 1 equivalent of water was used, exhibiting an optimal concentration. The turnover frequency toward β‐hydrocarboxylation was enhanced up to 38 s^−1^ under overpotential of 760 mV, which shows that reaction rate can be controlled by applied potential without the change in the product selectivity. We revealed that the water molecule is directly consumed as a proton source in β‐hydrocarboxylation by deuterium labeling experiments. Moreover, on the basis of the kinetic study, we suggested that the protonation and incorporation of CO_2_ on the benzylic position of styrene competitively occurs after the formation of the β‐carboxylate intermediate. Follow‐up studies on extending substrates to general unsaturated hydrocarbons such as aliphatic olefins are in progress. We expect that our strategy will aid understanding the role of proton sources in electrochemical platforms and further exploit the desired site‐selective carboxylation reactions. Furthermore, we envision that this method can be used in hybridizing the electrochemical reduction of CO_2_ and carbon fixation to long‐chain hydrocarbons as photosynthetic carbon cycle.^23^

## 4 Experimental Section

*Materials*: 5,5‐dimethyl‐pyrroline N‐oxide (DMPO), hydrocinnamic acid, 2‐phenylsuccinic acid, styrene, all styrene derivatives, tetrabutylammonium tetrafluoroborate (TBABF_4_), and tetramethylsilane (TMS) were purchased from TCI chemicals (Tokyo, Japan). (2,2,6,6‐Tetramethylpiperidin‐1‐yl)oxyl (TEMPO), chloroform‐*d* (CDCl_3_), and dimethyl sulfoxide‐*d*6 (DMSO‐*d*6) were purchased from Acros Organics (Geel, Belgium). Benzoquinone (BQ), iodomethane, and magnesium sulfate (MgSO_4_) were purchased from Sigma‐Aldrich (Milwaukee, WI, USA). Acetic acid, acetonitrile, *tert*‐butanol, *N*,*N*‐dimethylformamide (DMF), diethyl ether, hydrochloric acid (HCl), methanol, phenol, potassium carbonate (K_2_CO_3_), and sodium hydroxide (NaOH) were purchased from Daejung chemicals (Gyenonggido, Korea). DMF was dried with 4 Å molecular sieve, and all other chemicals were used as received. Purified deionized water (18.2 MΩ cm^−1^) was used in the procedures. Nickel foil (Ni, 0.1 mm thick), titanium foil (Ti, 0.127 mm thick), platinum foil (Pt, 0.025 mm thick), and magnesium foil (Mg, 0.25 mm thick) were purchased from Alfa Aesar (MA, USA).

*Electrochemical Analysis*: An undivided three‐electrode cell equipped with a gas line was used in all voltammetric measurements and electrolysis. Ni, Ti, and Pt with surface dimensions of 1 cm × 2.5 cm and Mg with surface dimensions of 1 cm × 3 cm were used as the working electrode and counter electrode, respectively. Prior to use, the metal foils were polished with sandpaper, cleaned with diluted HCl (aq.) and rinsed with distilled water. The reference electrode was Ag/Ag^+^ (0.01 m)/TBABF_4_ (0.1 m) in acetonitrile. For the electrolyte, TBABF_4_ (0.1 m) was put in dried DMF and saturated with CO_2_ by purging the gas for 1 h. Then, 4 mL of the solution was put into the reactor cell at a volume of 50 mL. The reactants were injected into the electrolyte, and the headspace of the cell was ventilated with CO_2_ gas for 0.5 h and closed tightly under CO_2_ at atmospheric pressure. All reactions were performed at room temperature under vigorous stirring of the electrolyte. After electrolysis, 1 mL of headspace gas was transferred by syringe for gas chromatography (GC) analysis. The solutions in the reactor cell were acidified with HCl (2 m, aq.) for 3 h and extracted with diethyl ether (3 × 20 mL). The organic layer was washed with distilled water, dried over MgSO_4_ and evaporated. The isolated products were dissolved in deuterated solvents for nuclear magnetic resonance (NMR) analysis or diethyl ether for gas chromatography‐mass spectrometry analysis.

*Methylation Procedure for GC‐MS Analysis*: Iodomethane (3 equivalents relative to the styrene reactant) and K_2_CO_3_ (2 equivalents relative to the styrene reactant) were mixed into the reaction solution immediately after electrolysis and stirred for 12 h under an argon atmosphere. The residues were extracted with diethyl ether (3 × 20 mL). The organic layer was washed with distilled water, dried with MgSO_4_ and concentrated under reduced pressure.

*Analytical Methods*: NMR spectra were recorded on a JEOL 400 MHz NMR spectrometer (JeolJMN‐LA400) or Bruker 600 MHz NMR spectrometer (Bruker Avance 600) at room temperature. Chemical shifts were reported in parts per million (ppm) downfield of TMS (δ = 0.00 ppm). Products were dissolved into 0.7 mL of DMSO‐*d*6 or CDCl_3_. A potentiostat (CHI 760E, CH Instruments) was used for voltammetric measurements and bulk electrolysis. All potentials were controlled against the reference electrode and recorded after IR compensation. The quantitative measurement of the gas phase from the headspace of the electrochemical cell was performed by GC (PerkinElmer, NARL8502 Model 4003). Liquid‐ and solid‐phase products dissolved in the organic solvents were detected by GC‐MS (Agilent 5977) using an automatic liquid sampler.

## Conflict of Interest

The authors declare no conflict of interest.

## Supporting information

Supporting Information
