# Supplementary material for: Electrochemical β‐Selective Hydrocarboxylation of Styrene Using CO2 and Water
Source: Adv Sci (Weinh). 2019 Dec 17;7(3):1900137. doi: 10.1002/advs.201900137 (PMC7001630; doi:10.1002/advs.201900137)
Supplement: Supplementary file 1 — Supporting Information [file ADVS-7-1900137-s001.pdf]

Copyright WILEY-VCH Verlag GmbH & Co. KGaA, 69469 Weinheim, Germany, 2019.

ADVANCED SCIENCE
Open Access

Supporting Information

for *Adv. Sci.,* DOI: 10.1002/advs.201900137

Electrochemical #-Selective Hydrocarboxylation of Styrene Using CO_2 and Water

*Younghye Kim, Gyeong Do Park, Mani Balamurugan, Jiwon Seo, Byoung Koun Min, and Ki Tae Nam**

WILEY-VCH

Copyright WILEY-VCH Verlag GmbH & Co. KGaA, 69469 Weinheim, Germany, 2019.

Supporting Information

Electrochemical *β*-Selective Hydrocarboxylation of Styrene Using CO_2 and Water

*Younghye Kim[1,2], Gyeong Do Park[1], Mani Balamurugan[1], Jiwon Seo[3], Byoung Koun Min[2] and Ki Tae Nam[1]**

[1]Department of Materials Science and Engineering, Seoul National University, Seoul 151-744, Republic of Korea
[2]Clean Energy Research Center, Korea Institute of Science and Technology, 5 Hwarang-ro 14-gil, Seongbuk-gu, Seoul 02792, Republic of Korea
[3]Department of Chemistry, School of Physics and Chemistry, Gwangju Institute of Science and Technology, Gwangju 61005, Republic of Korea
*Correspondence to: nkitae@snu.ac.kr

Contents

1. Turnover frequency calculation

2. NMR results of the deuterium-labeling experiment

3. GC-MS results of the deuterium-labeling experiment

4. Characterization of products

5. NMR spectra

6. Supporting figures and tables

7. References

1. Turnover frequency (TOF) calculation

The TOF (Table 1) was calculated from the following equation:[1]

$$\mathrm{TOF} = \frac{\text{Current density} \times \mathrm{N_A} \times \text{Yield}}{\text{Faraday constant} \times \text{involved electron number} \times \text{Ative site density}}$$

We assumed that all the metal atoms on the surface of the electrode are electrochemically active. Thus, the average surface atom density of the polycrystalline nickel electrode was used as the active site density. The surface atom density was calculated based on the atomic volume of nickel.[2]

$$\text{Surface atom density} = \left(\frac{\mathrm{N_A}}{\text{atomic volume}}\right)^{\frac{2}{3}} = \left(\frac{6.022 \times 10^{23}}{6.67\ \mathrm{cm^3}}\right)^{\frac{2}{3}} = 2.01 \times 10^{15}\ \mathrm{cm^2}$$

For example, at an overpotential of 320 mV (entry 3, Table 1), the TOF of *β*-hydrocarboxylation was calculated as follows.

$$\mathrm{TOF} = \frac{10\ (mA \cdot cm^{-2}) \times 6.022 \times 10^{23} \times 65(\%)}{96485\ (\mathrm{s \cdot A} \cdot mol^{-1}) \times 2 \times 2.01 \times 10^{15}(cm^2)}$$
$$= \frac{10 \times 10^{-3} \times 6.022 \times 10^{23} \times 0.65}{96485 \times 2 \times 2.01 \times 10^{15}}\ (s^{-1}) = 10\, s^{-1}$$

2. NMR results of the deuterium-labeling experiment

Top: ^{1}H NMR of deuterium-labeled hydrocinnamic acid (400 MHz, $CDCl_3$) δ 7.31 -7.27 (m, 2H), 7.24 - 7.20 (m, 3H), 2.94 (t, *J* = 7.2 Hz, 2H), 2.55 (t, *J* = 7.6 Hz, 2H)

Bottom: ^{1}H NMR of unlabeled hydrocinnamic acid (400 MHz, $CDCl_3$) δ 7.28 -7.24 (m, 2H), 7.20 - 7.16 (m, 3H), 2.92 (t, *J* = 8.0 Hz, 2H), 2.68 (t, *J* = 8.0 Hz, 2H)

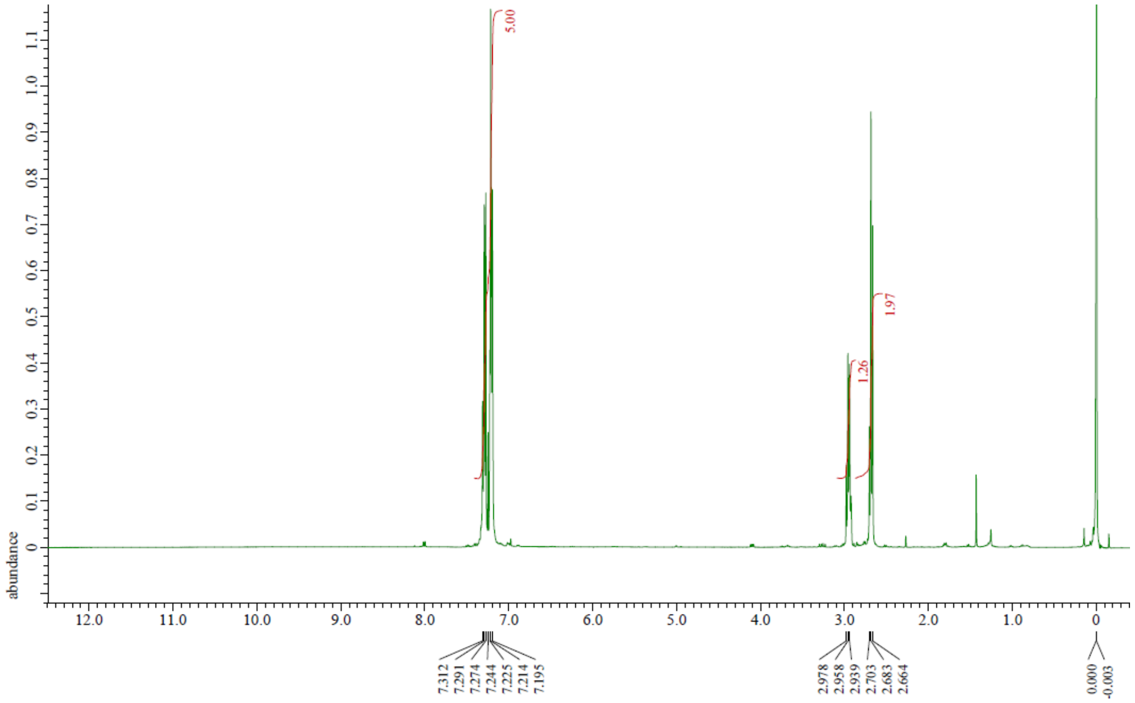

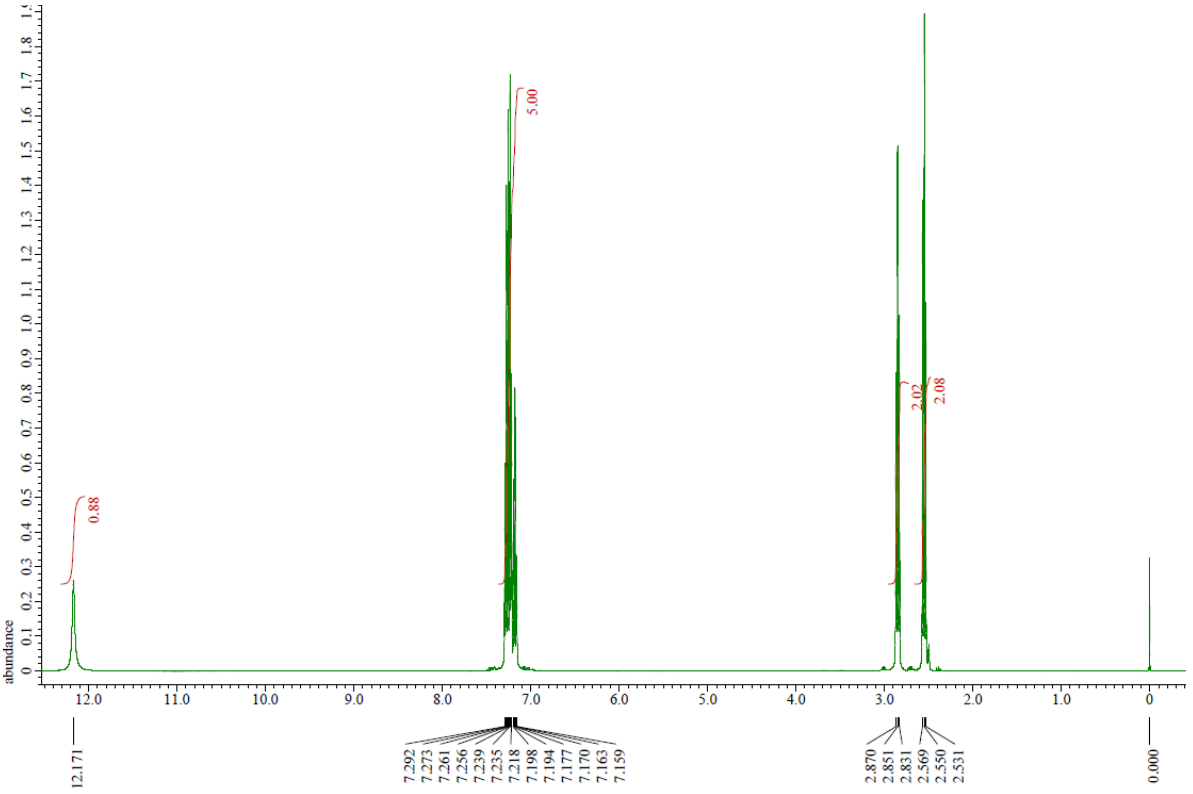

Magnified ^{1}H NMR spectra (δ 3.10 - 2.50) of deuterium-labeled (top) and unlabeled (bottom) hydrocinnamic acid

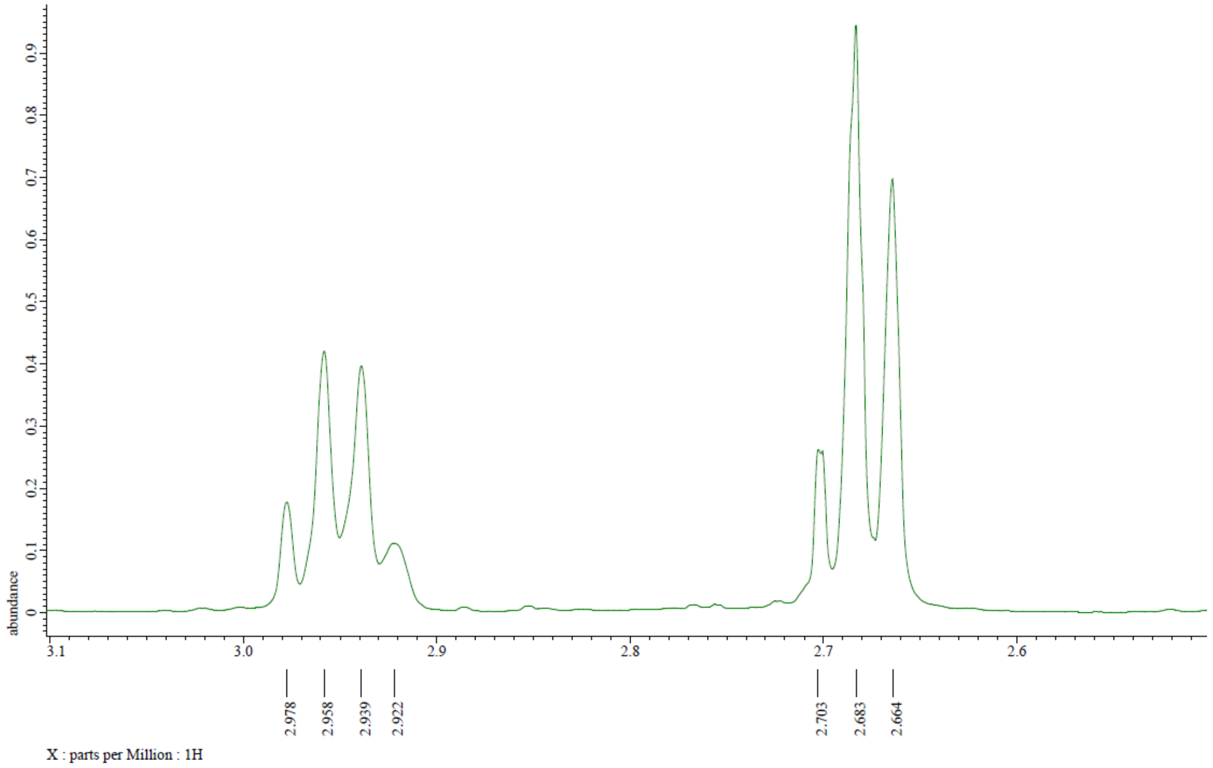

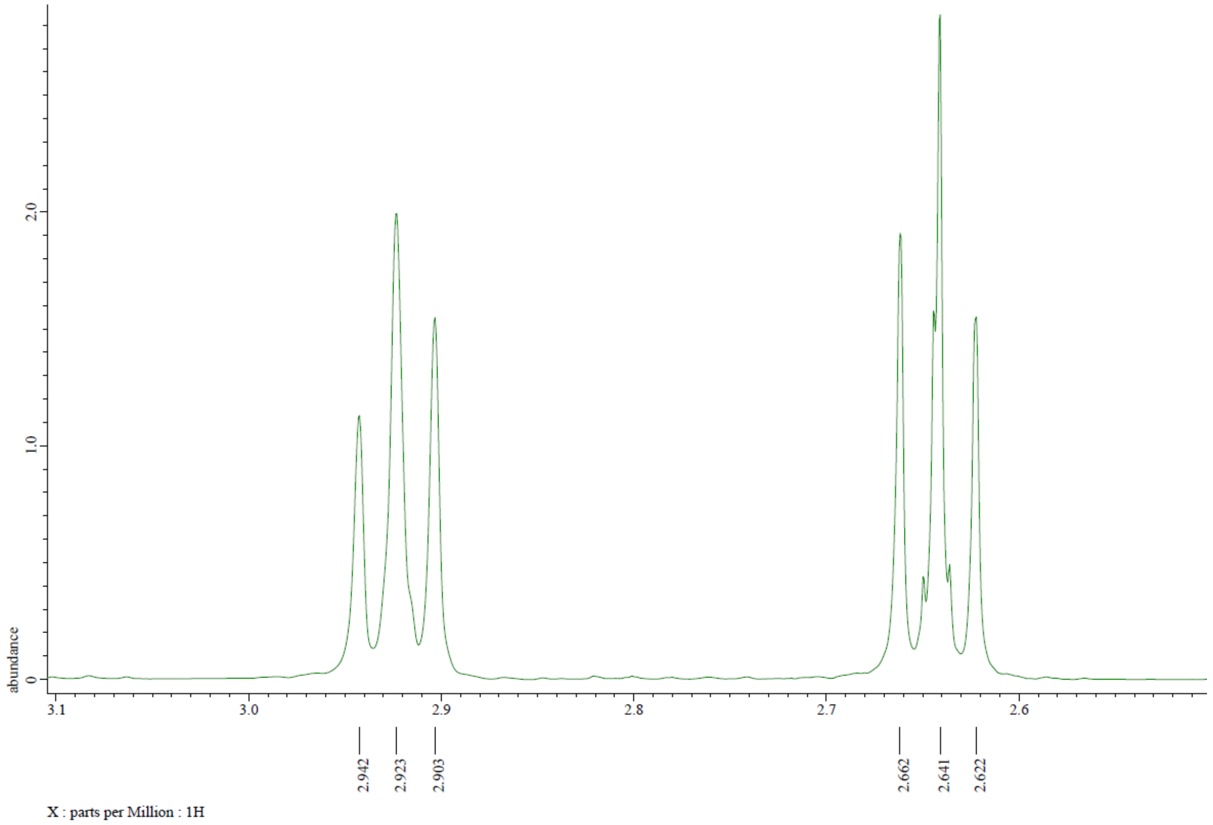

3. GC-MS results of the deuterium-labeling experiment

Reaction conditions: Electrolysis of styrene (0.1 M) with deuterium oxide (D_2O, 0.1 M) in 4 mL of CO_2-saturated DMF and $TBABF_4$ (0.1 M) electrolyte was conducted on a Ni cathode-Mg anode for total charge of 20 C at room temperature and under atmospheric pressure of CO_2. Then, iodomethane (0.3 M) and K_2CO_3 (0.2 M) were mixed into the reaction solution and stirred for 12 h under an argon atmosphere. The residues were treated with HCl (2 M, aq) and extracted with diethyl ether (3 × 20 mL). The organic layer was washed with distilled water, dried with $MgSO_4$, and evaporated. The products were dissolved in diethyl ether (1 mL) and injected through a liquid sample auto-injector for gas chromatography analysis. All labeling experiments were replicated twice. The measured chromatogram is as follows.

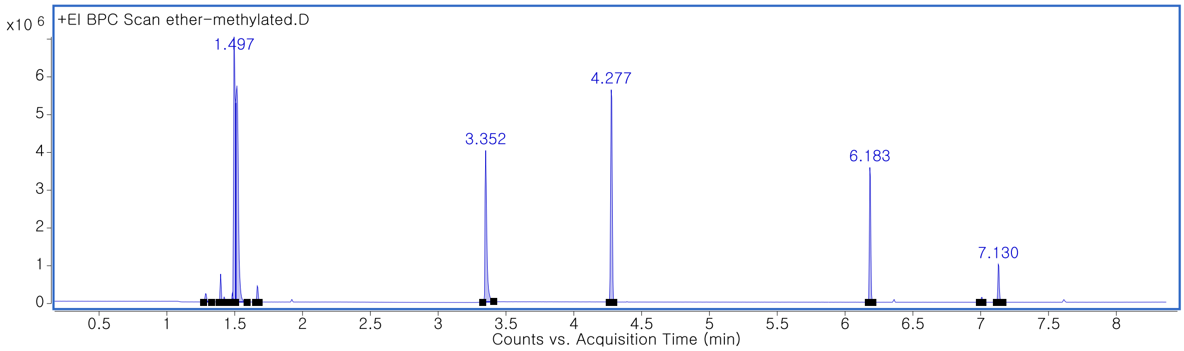

Product lists (acquisition time): Diethyl ether (1.497), DMF (3.352), styrene (4.277), dimethyl-2-phenylsuccinate (6.183) and methyl-3-phenylpropanoate (7.130)

Mass spectrum of dimethyl-2-phenylsuccinate:

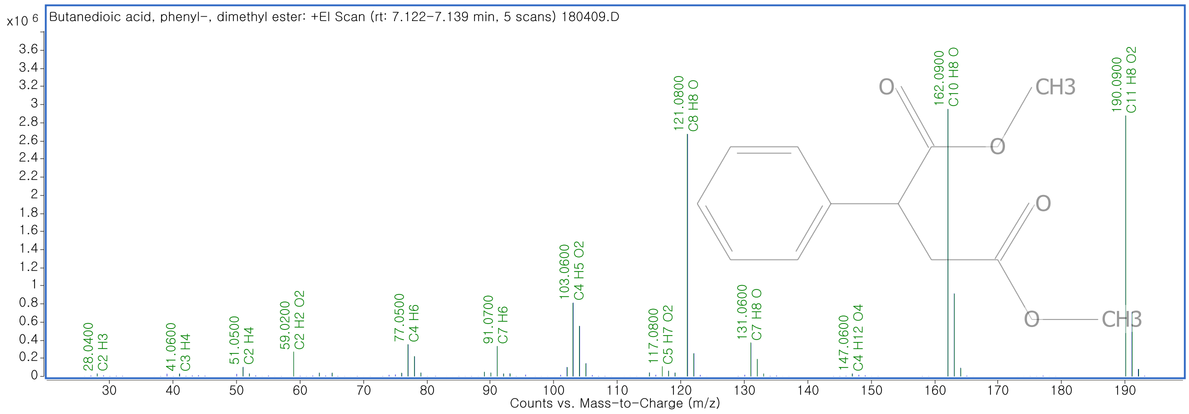

4. Characterization of products

Reaction conditions: Electrolysis of reactant styrenes (0.1 M) with water (0.1 M) in 4 mL of CO_2-saturated DMF and $TBABF_4$ (0.1 M) electrolyte was conducted on a Ni cathode-Mg anode for total charge of 20 C at room temperature and under atmospheric pressure of CO_2. Then, reactants were treated with HCl (2 M, aq) for 3 h and extracted with diethyl ether (3 × 20 mL). The organic layer was washed with distilled water and evaporated. The obtained products were re-dissolved in diethyl ether (10 mL) and extracted with of 1 M NaOH (3 × 20 mL). The aqueous solution was washed with diethyl ether, acidified with HCl (2 M, aq) and extracted with diethyl ether (3 × 20 mL). The organic layer was washed with distilled water, dried with $MgSO_4$, and evaporated to give the final product.

2-Phenylsuccinic acid (**1**)

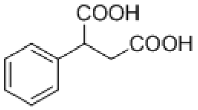

^{1}H NMR (400 MHz, DMSO-*d6*) δ 12.37 (br s, 2H), 7.35 - 7.24 (m, 5H), 3.94 (q, *J* = 4.8 Hz, 1H), 3.00 (dd, *J* = 17.2, 10.4 Hz, 1H), 2.57 (dd, *J* = 16.8, 5.2 Hz, 1H). ^{13}C NMR (100 MHz, DMSO-*d*6) δ 174.6, 173.2, 139.2, 129.2, 128.3, 127.7, 47.4, 38.0.

Hydrocinnamic acid (**2**)

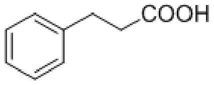

^{1}H NMR (400 MHz, DMSO-*d6*) δ 12.17 (s, 1H) 7.29 - 7.22 (m, 2H), 7.20 - 7.16 (m, 3H), 2.85 (t, J = 8.0 Hz, 2H), 2.55 (t, *J* = 8.0 Hz, 2H). ^{13}C NMR (100 MHz, DMSO-*d*6) δ 174.3, 141.4, 128.8, 128.7, 126.5, 35.8, 30.9. HRMS (m/z) $[M-H]^-$ Calcd. for $C_9H_9O_2$, 149.0681; Found, 149.0400.

3-(4-Methoxyphenyl)propanoic acid (**2a**)

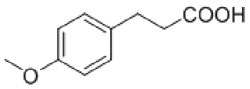

^{1}H NMR (400 MHz, $CDCl_3$) δ 7.14 (d, J = 8.8 Hz, 2H), 6.84 (d, *J* = 8.4 Hz, 2H), 3.79 (s, 3H), 2.90 (t, *J* = 7.6 Hz, 2H), 2.64 (t, *J* = 7.8 Hz, 2H). ^{13}C NMR (150 MHz, $CDCl_3$) δ 178.7, 158.3, 132.4, 129.4, 114.1, 55.4, 36.01, 29.9. HRMS (m/z) $[M-H]^-$ Calcd. for $C_{10}H_{11}O_3$, 179.0786; Found, 179.0525.

3-(p-Tolyl)propanoic acid (**2b**)

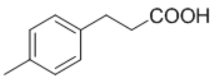

^{1}H NMR (400 MHz, $CDCl_3$) δ 7.10 (s, 4H), 2.92 (t, *J* = 8.4 Hz, 2H), 2.66 (t, *J* = 8.0 Hz, 2H), 2.32 (s, 3H). ^{13}C NMR (150 MHz, $CDCl_3$) δ 178.7, 137.1, 135.9, 129.2, 128.1, 35.7, 30.2, 21.0. HRMS (m/z) $[M-H]^-$ Calcd. for $C_{10}H_{11}O_2$, 163.0837; Found, 163.0601.

3-(4-Fluorophenyl)propanoic acid (**2c**)

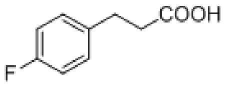

^{1}H NMR (400 MHz, $CDCl_3$) δ 7.16 (t, *J* = 6.8 Hz, 2H), 6.97 (t, *J* = 8.4 Hz, 2H), 2.92 (t, *J* = 8.4 Hz, 2H), 2.68 (t, *J* = 7.6 Hz, 2H). ^{13}C NMR (150 MHz, $CDCl_3$) δ 178.3, 162.3, 160.7, 135.8, 129.7, 129.7, 115.4, 115.2, 35.7, 29.8. HRMS (m/z) $[M-H]^-$ Calcd. for $C_9H_8FO_2$, 167.0587; Found, 167.0389.

3-(3-Fluorophenyl)propanoic acid (**2d**)

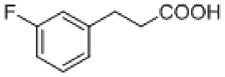

^{1}H NMR (400 MHz, $CDCl_3$) δ 7.28 - 7.22 (m, 1H), 6.99 - 6.89 (m, 3H), 2.93 (t, *J* = 7.6 Hz, 2H), 2.65 (t, *J* = 7.6 Hz, 2H). ^{13}C NMR (150 MHz, $CDCl_3$) δ 177.8, 163.7, 162.1, 142.7, 130.0, 123.9, 115.3, 113.4, 35.1, 30.3. HRMS (m/z) $[M-H]^-$ Calcd. for $C_9H_8FO_2$, 167.0587; Found, 167.0400.

3-(4-Chlorophenyl)propanoic acid (**2e**)

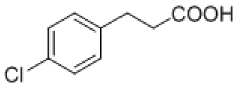

^{1}H NMR (400 MHz, $CDCl_3$) δ 7.28 - 7.23 (m, 4H), 2.97 (t, *J* = 7.8 Hz, 2H), 2.70 (t, *J* = 7.8 Hz, 2H). ^{13}C NMR (150 MHz, $CDCl_3$) δ 178.2, 140.2, 129.0, 128.6, 128.3, 35.5, 30.6. HRMS (m/z) $[M-H]^-$ Calcd. for $C_9H_8ClO_2$, 183.0291; Found, 183.0190.

4-Vinylbenzoic acid (**3**)

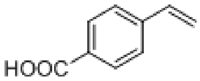

^{1}H NMR (400 MHz, $CDCl_3$) δ 8.07 (d, *J* = 8.0 Hz, 2H), 7.49 (d, *J* = 8.4 Hz, 2H), 6.77 (dd, *J* = 17.6, 10.8 Hz, 1H), 5.89 (d, *J* = 17.6 Hz, 1H), 5.41 (d, *J* = 11.2 Hz, 1H). ^{13}C NMR (100 MHz, $CDCl_3$) δ 171.9, 142.8, 136.0, 130.6, 128.4, 126.2, 117.0. HRMS (m/z) $[M-H]^-$ Calcd. for $C_9H_7O_2$, 148.0524; Found, 148.0299.

5. NMR spectra

(1) NMR spectra of electrolyte with styrene substrates before the electrolysis

To identify potential impurities comes from the reactant chemicals and solvents, NMR spectrum of electrolyte with styrene substrates were obtained before the electrolysis. DMF and $TBABF_4$ (0.1 M) electrolyte with styrene substrates (0.1 M) were treated with HCl (2 M, aq) for 3 h and extracted with diethyl ether (3 × 20 mL). The organic layer was washed with distilled water, dried with $MgSO_4$ and evaporated. The isolated chemicals were dissolved in $CDCl_3$ for NMR analysis.
On the basis of the following NMR spectra, we distinguished the carboxylated product peaks from the residual impurity and substrate peaks. The unknown peaks may come from the chemical stabilizers and the solvent impurities.

Substrate : 4-Methoxystyrene

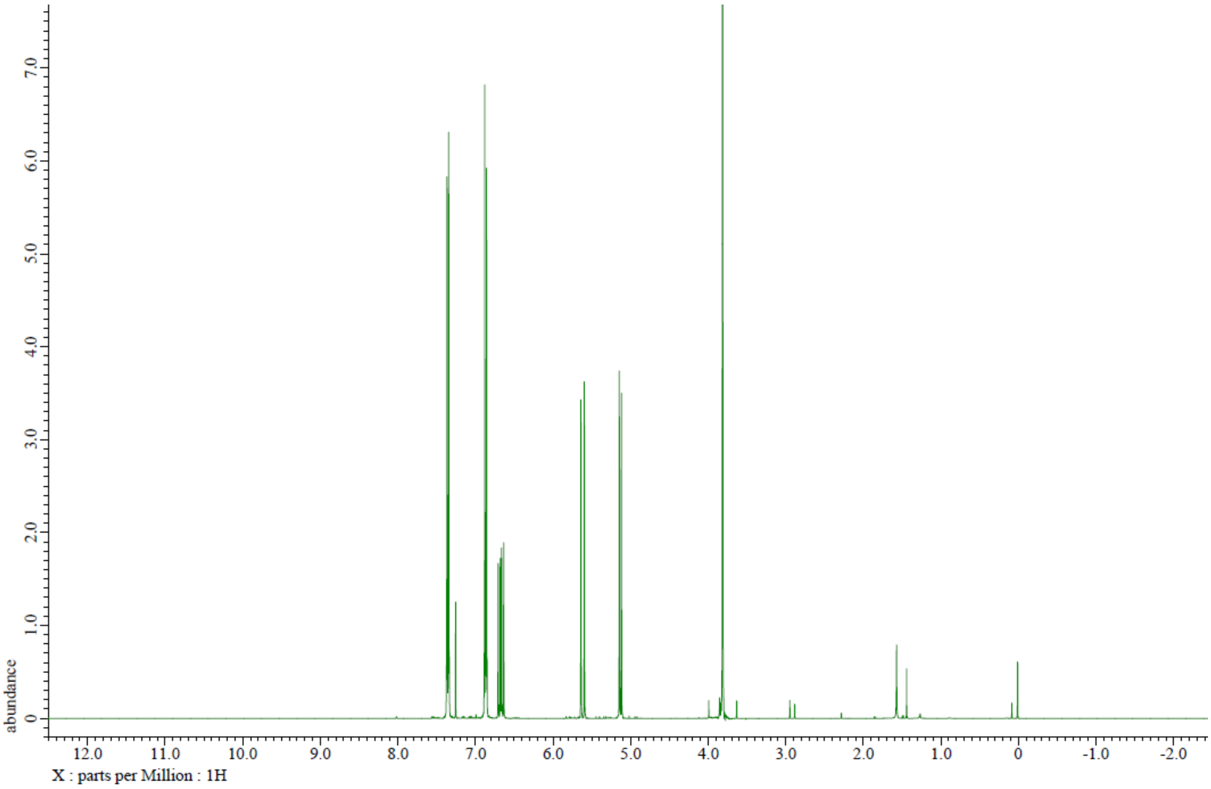

Substrate : 4-Methylstyrene

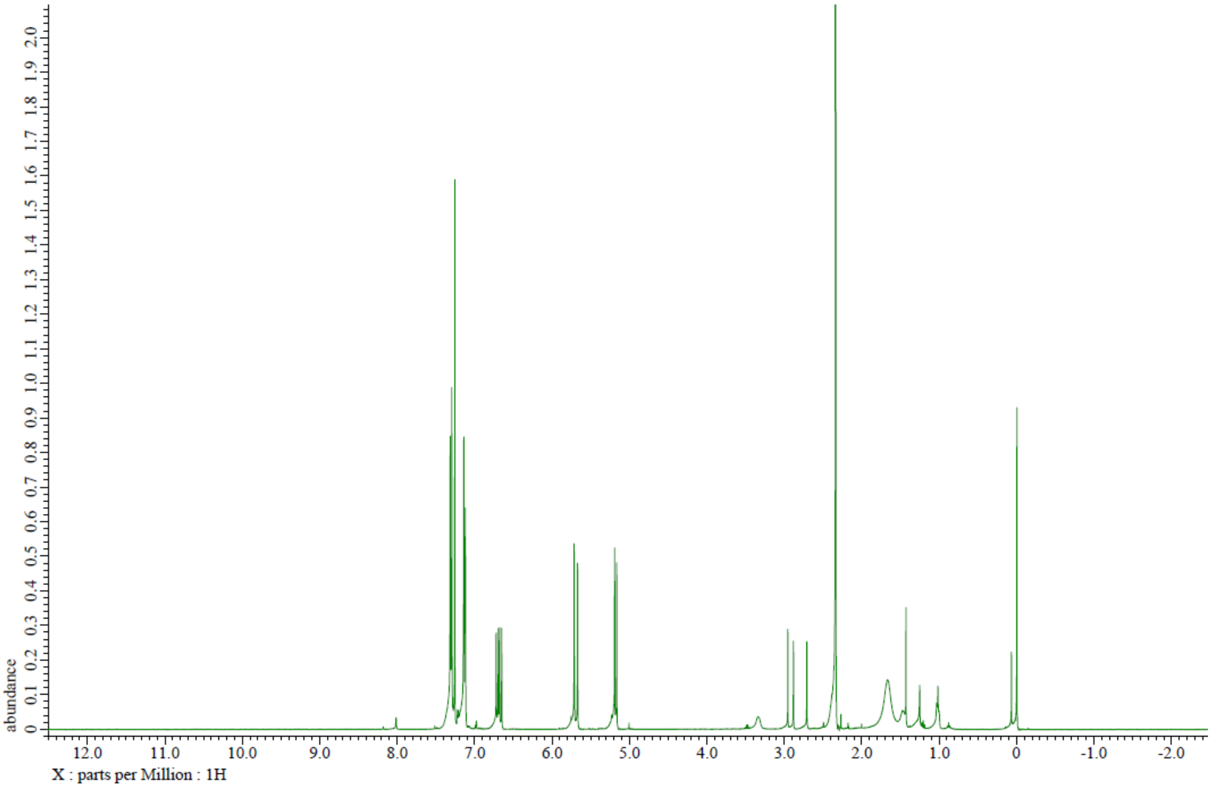

Substrate : 4-Fluorostyrene

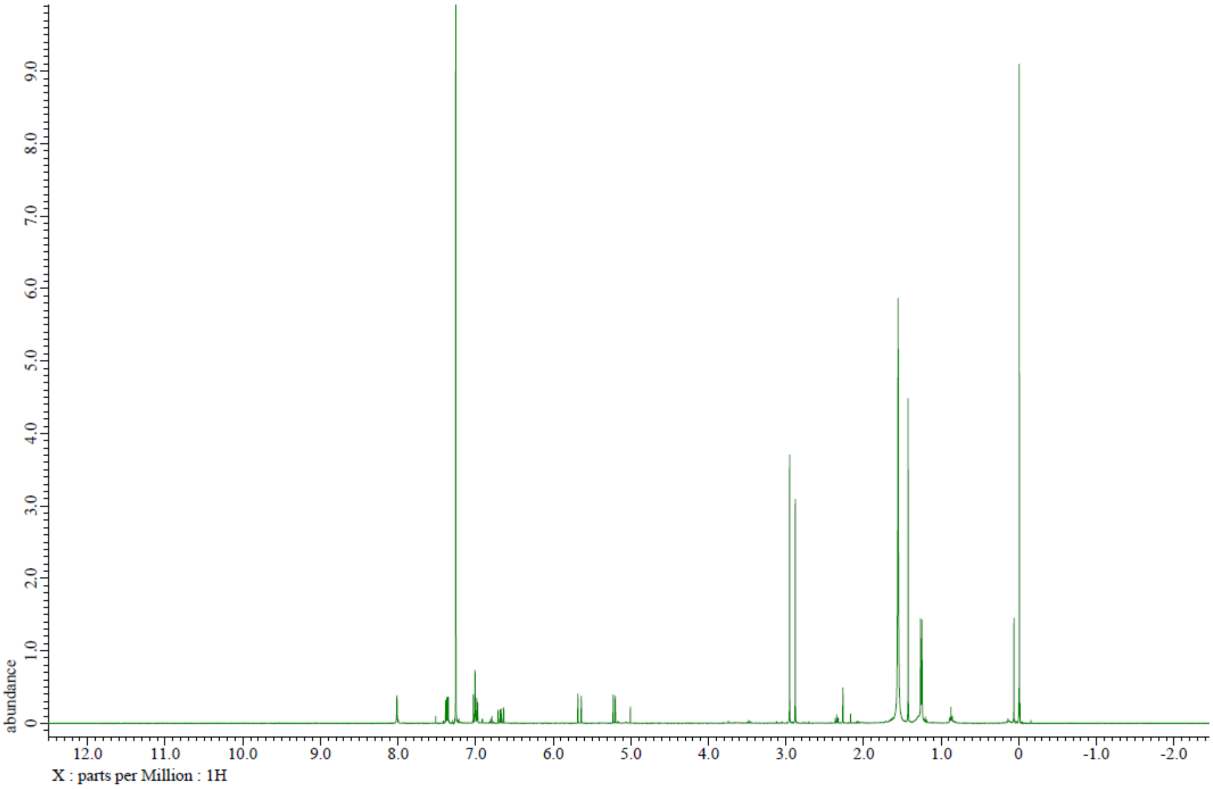

Substrate : 3-Fluorostyrene

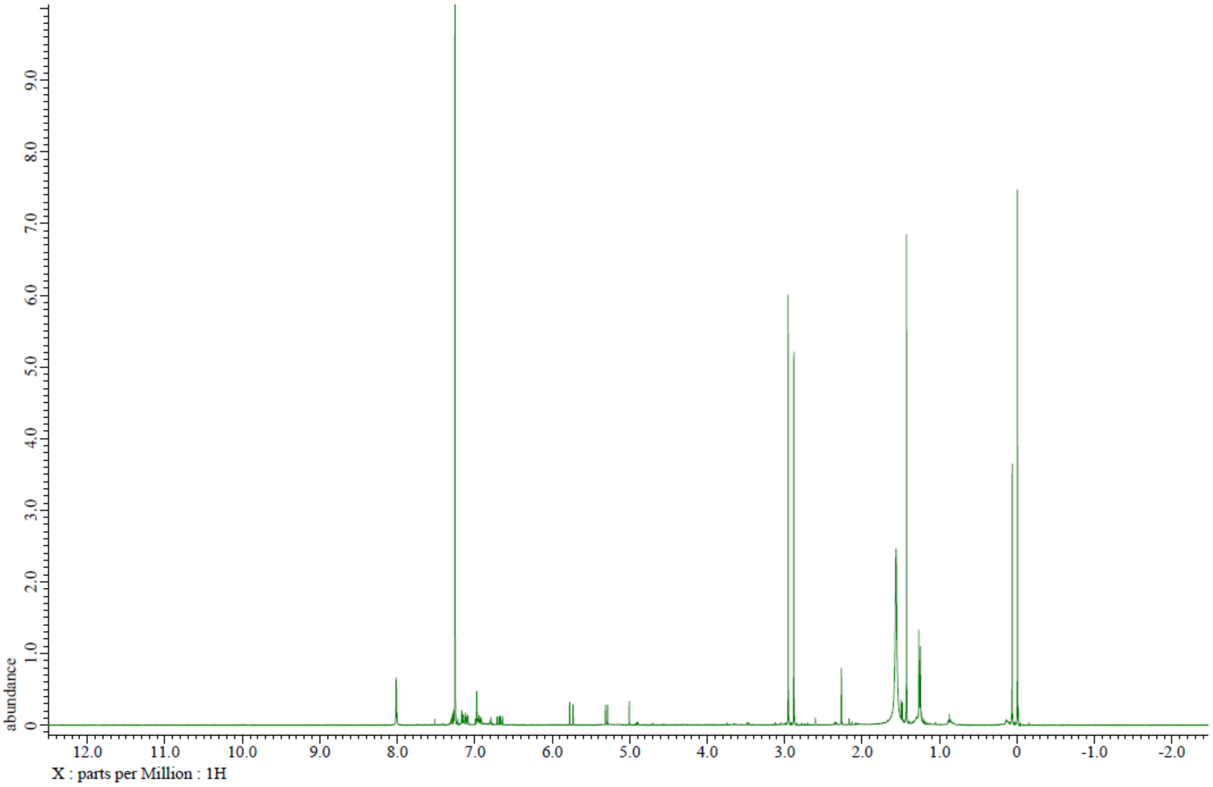

Substrate : 4-Chlorostyrene

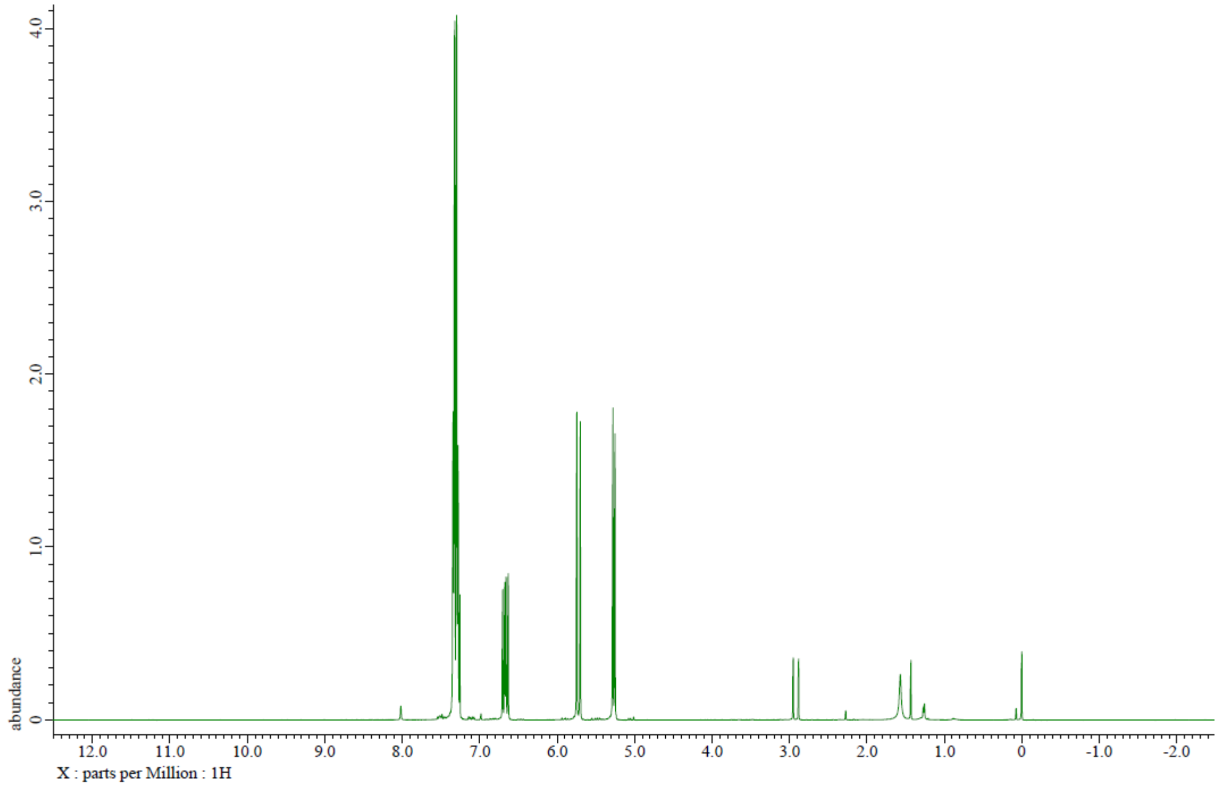

Substrate : 4-(Trifluoromethyl)styrene

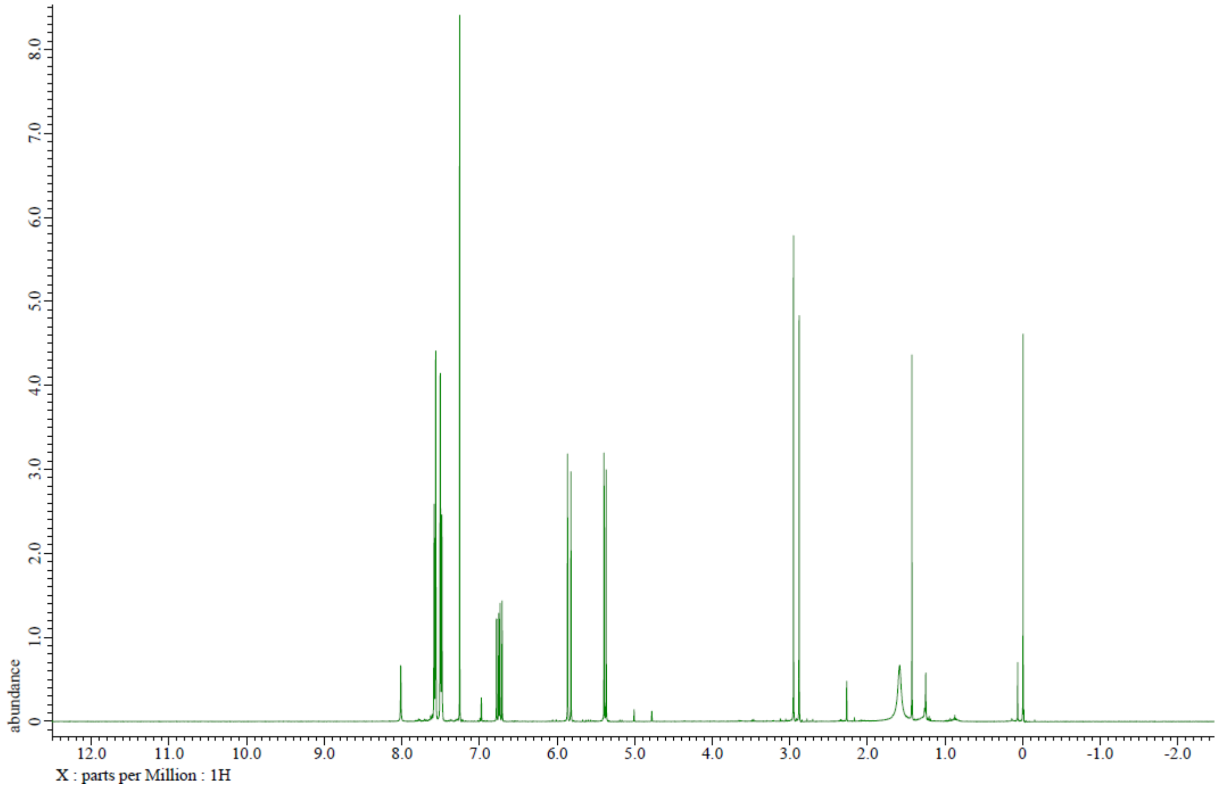

(2) NMR spectra of the carboxylated styrenes

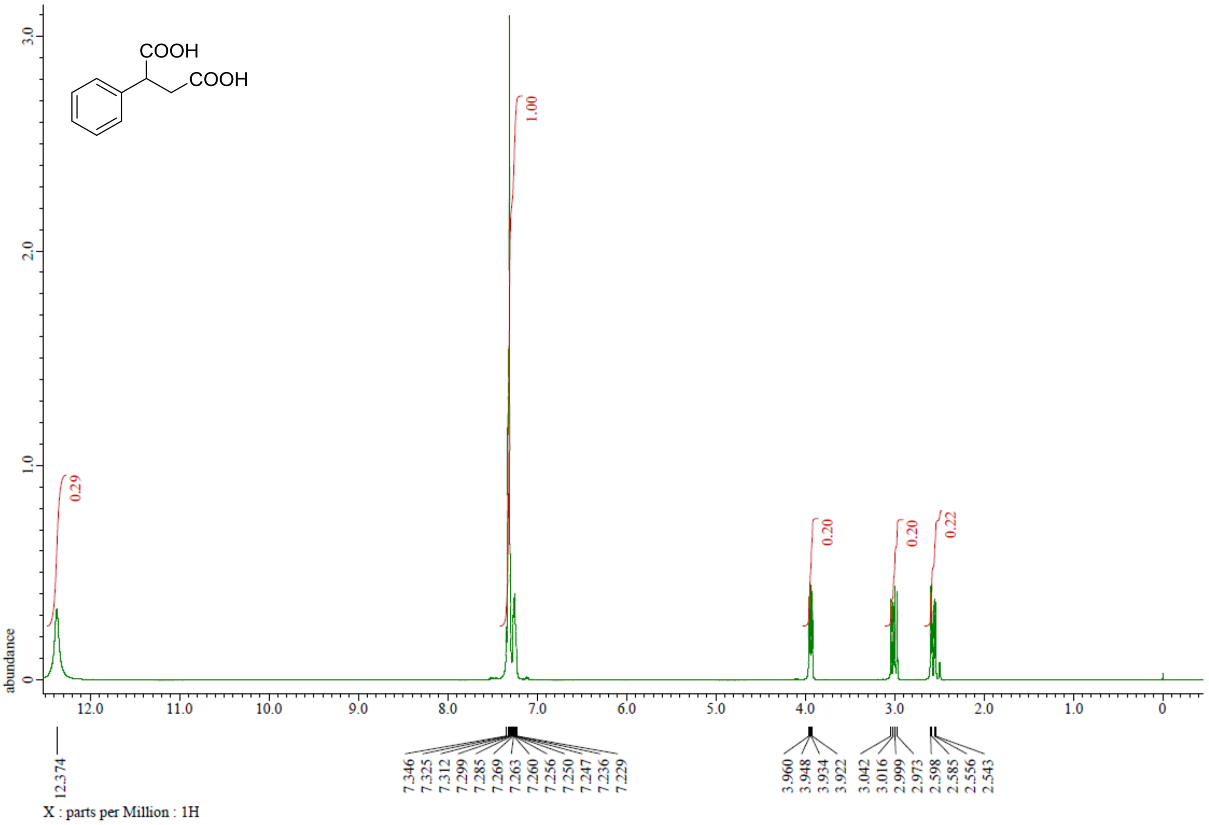

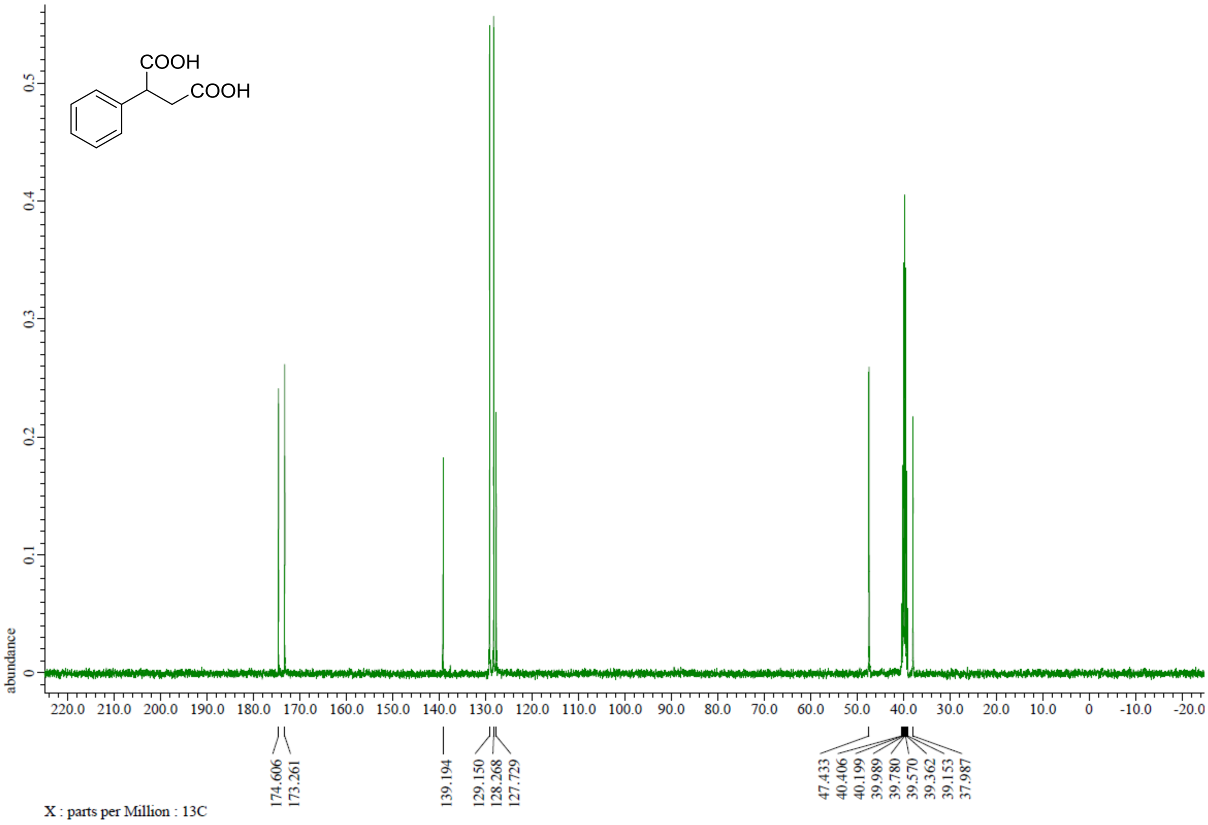

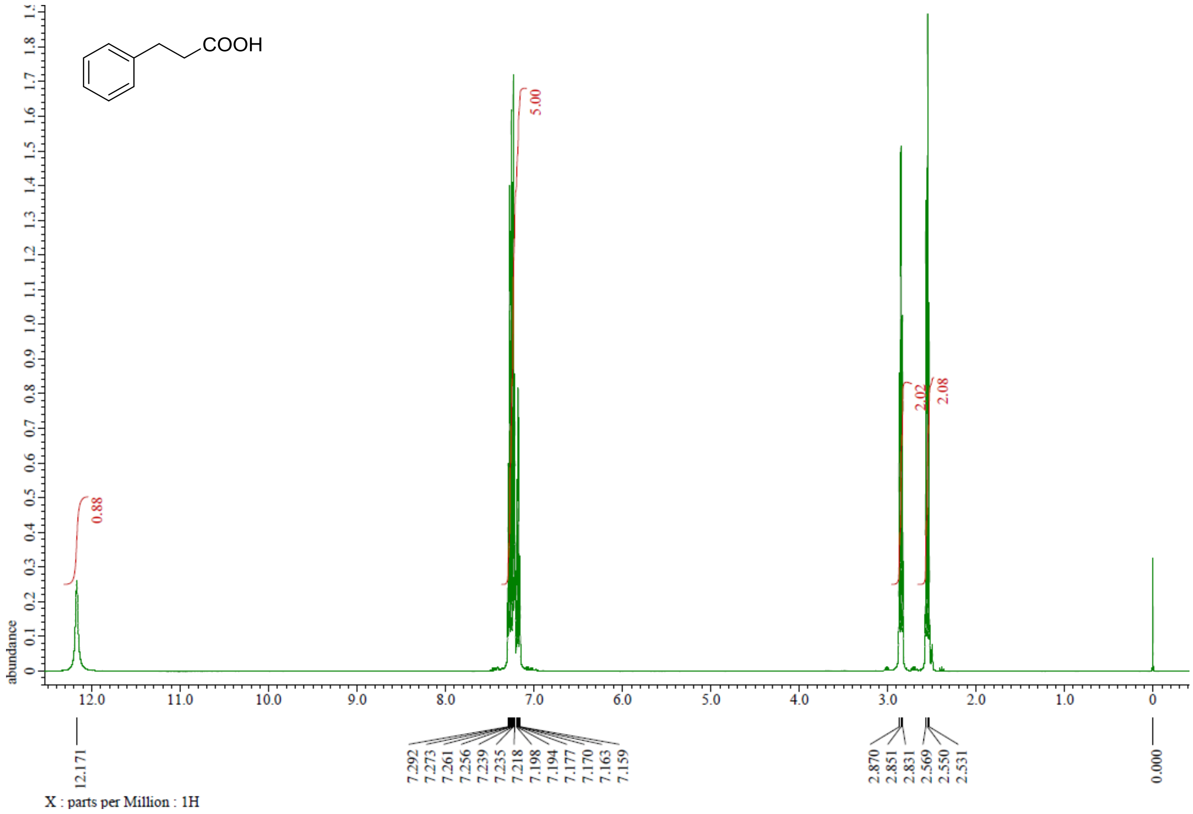
COOH
5.00
0.88
2.02
2.08
abundance
12.171
7.292 7.273 7.261 7.256 7.239 7.235 7.218 7.198 7.194 7.177 7.170 7.163 7.159
2.870 2.851 2.831 2.569 2.550 2.531
0.000
X : parts per Million : 1H

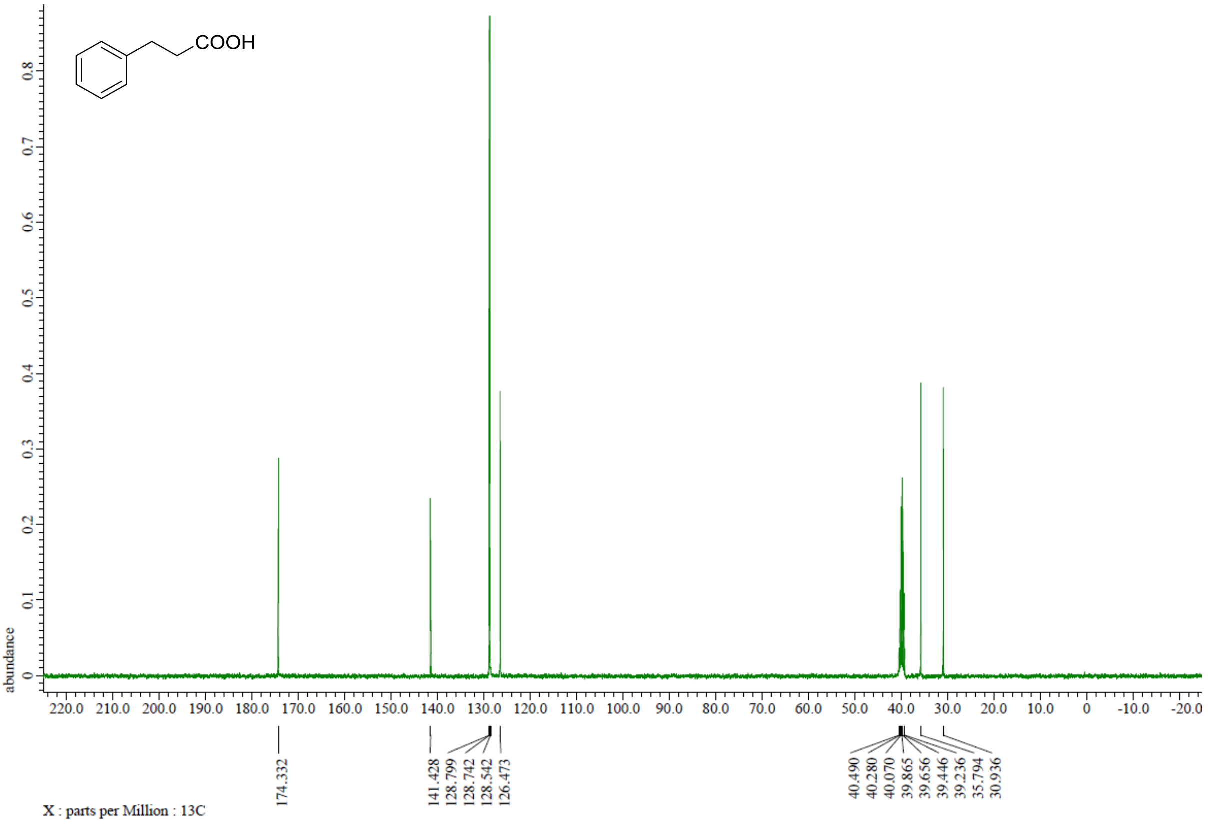
COOH
abundance
174.332
141.428 128.799 128.742 128.542 126.473
40.490 40.280 40.070 39.865 39.656 39.446 39.236 35.794 30.936
X : parts per Million : 13C

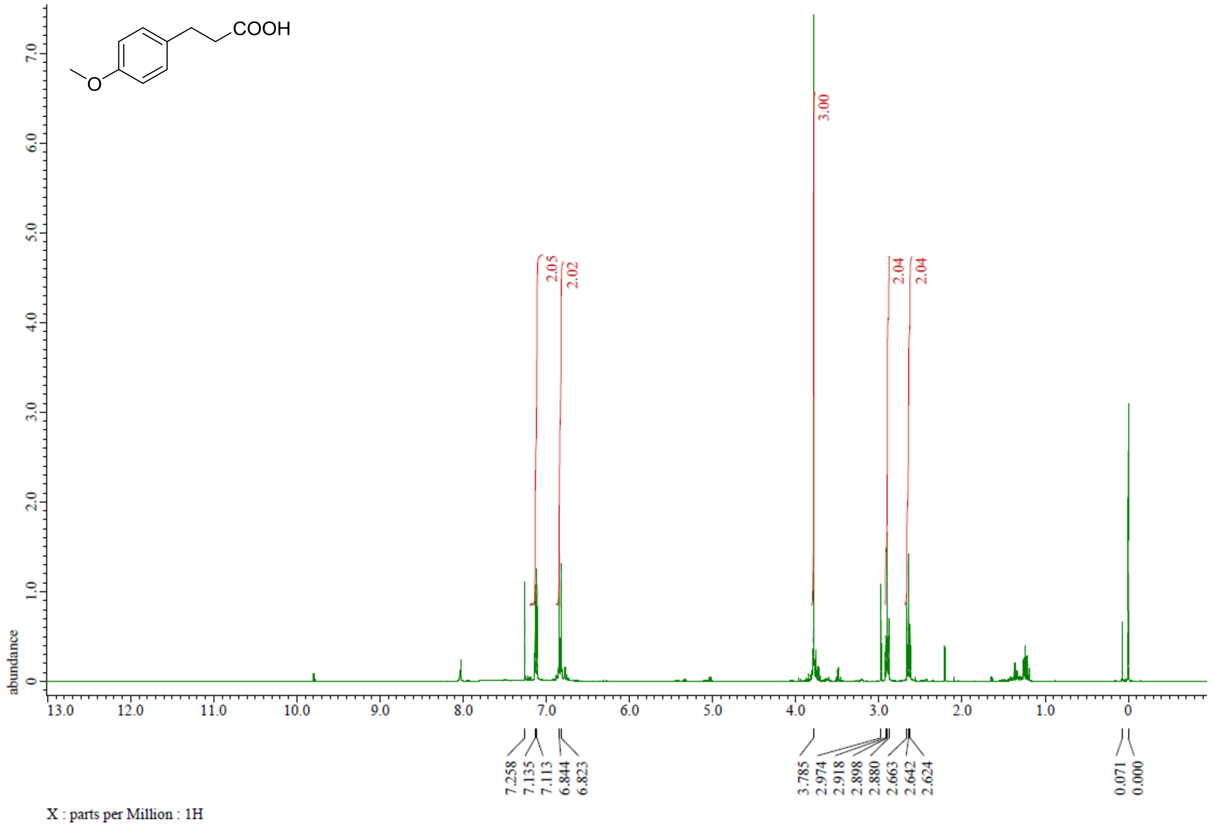
COOH
O
abundance
3.00
2.05
2.02
2.04
2.04
7.258
7.135
7.113
6.844
6.823
3.785
2.974
2.918
2.898
2.880
2.663
2.642
2.624
0.071
0.000
X : parts per Million : 1H

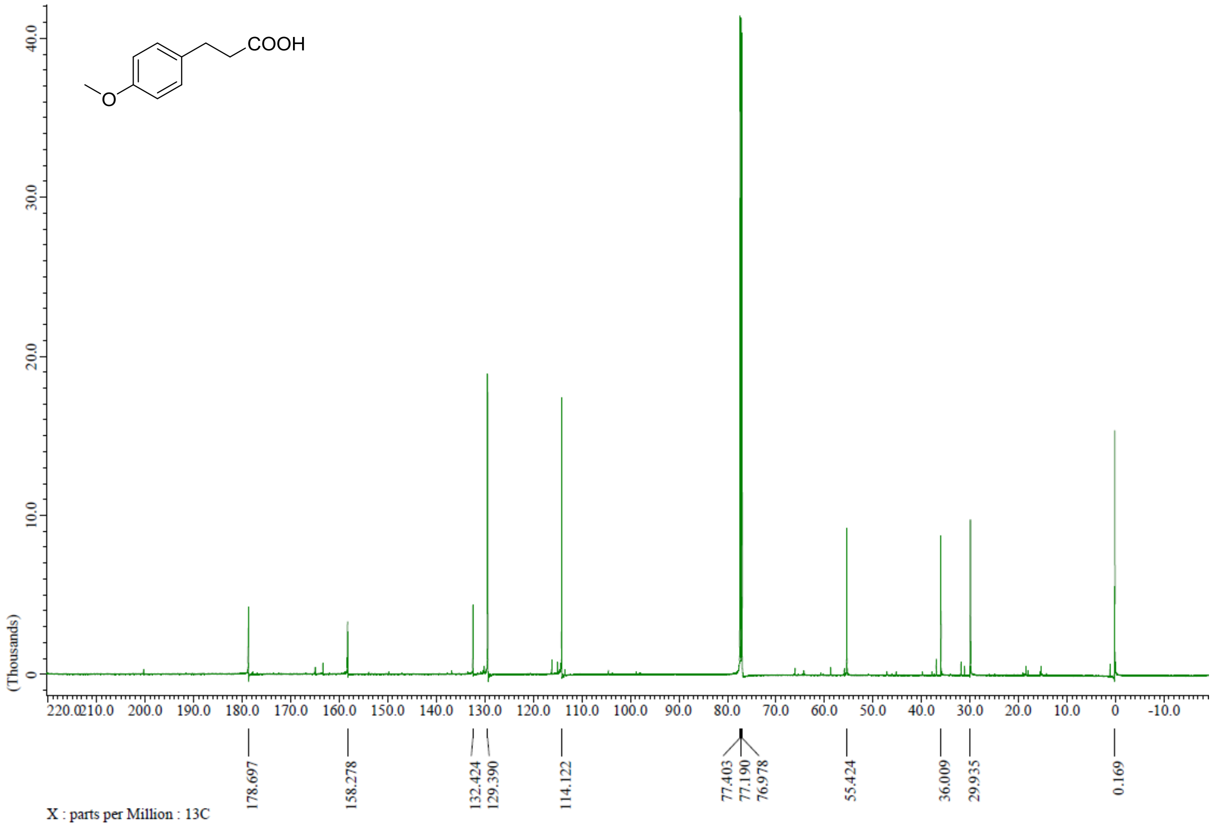
COOH
O
(Thousands)
178.697
158.278
132.424
129.390
114.122
77.403
77.190
76.978
55.424
36.009
29.935
0.169
X : parts per Million : 13C

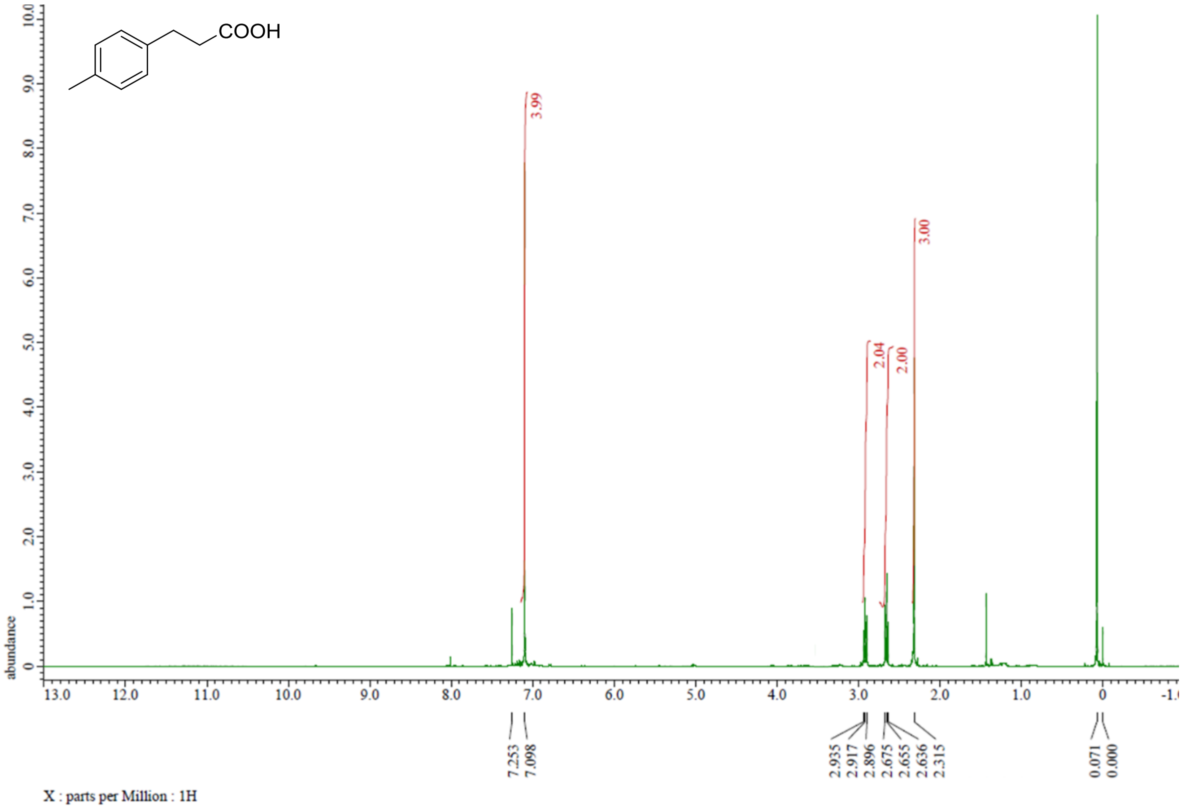
COOH
abundance
3.99
2.04
2.00
3.00
7.253
7.098
2.935
2.917
2.896
2.675
2.655
2.636
2.315
0.071
0.000
X : parts per Million : 1H

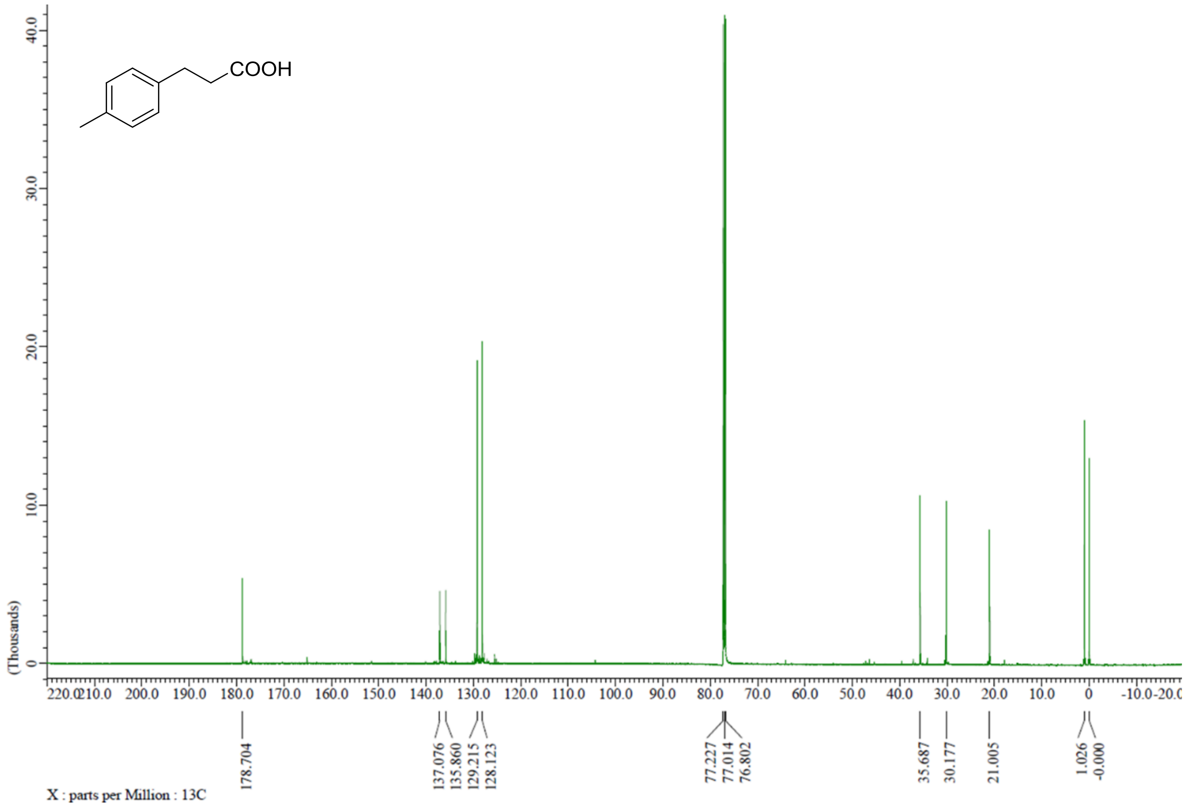
COOH
(Thousands)
178.704
137.076
135.860
129.215
128.123
77.227
77.014
76.802
35.687
30.177
21.005
1.026
-0.000
X : parts per Million : 13C

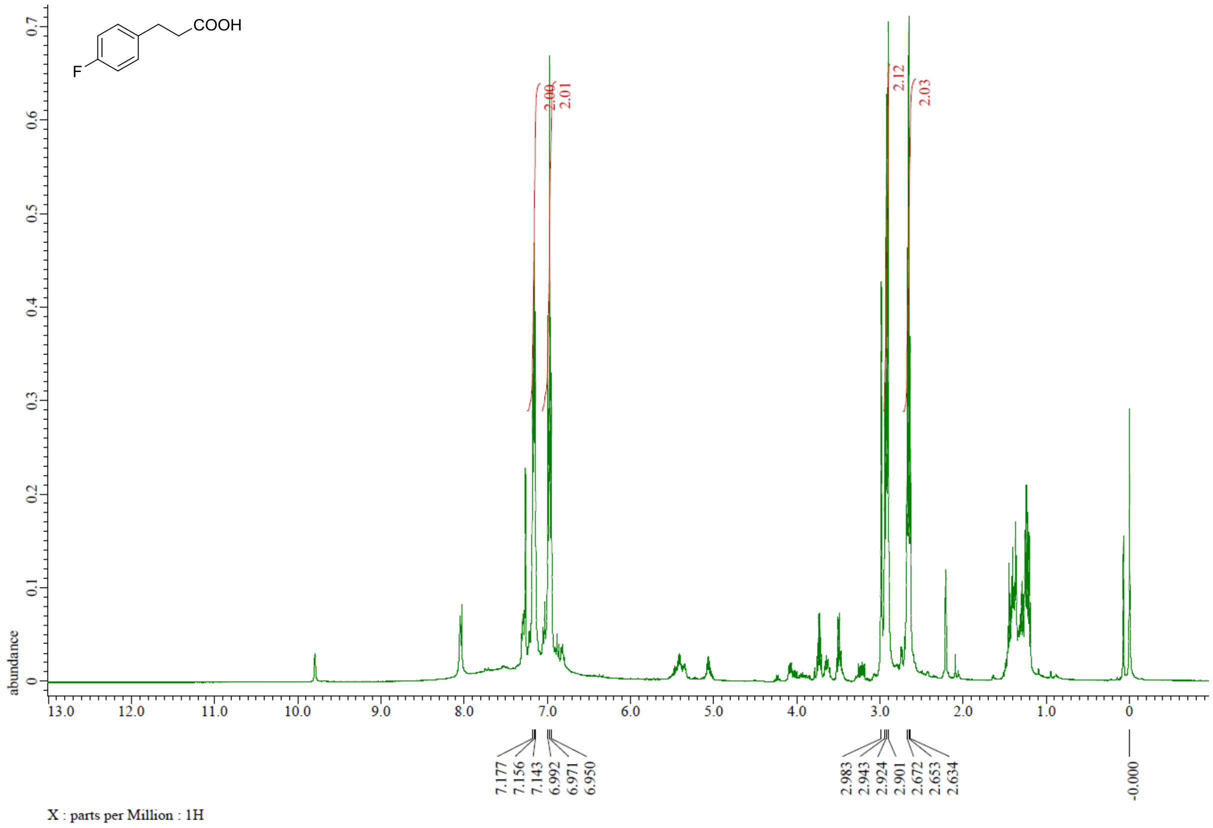
COOH
F
abundance
2.00
2.01
2.12
2.03
7.177
7.156
7.143
6.992
6.971
6.950
2.983
2.943
2.924
2.901
2.672
2.653
2.634
-0.000
X : parts per Million : 1H

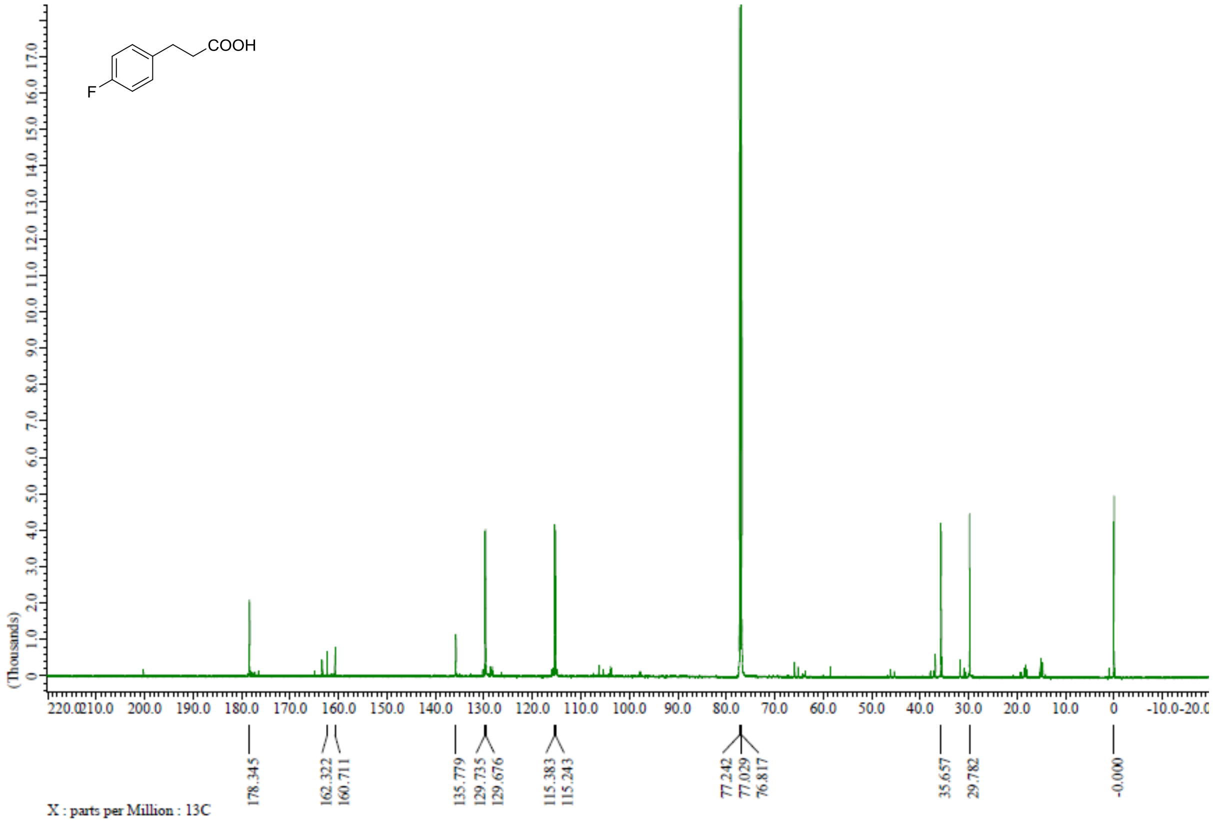
COOH
F
(Thousands)
178.345
162.322
160.711
135.779
129.735
129.676
115.383
115.243
77.242
77.029
76.817
35.657
29.782
-0.000
X : parts per Million : 13C

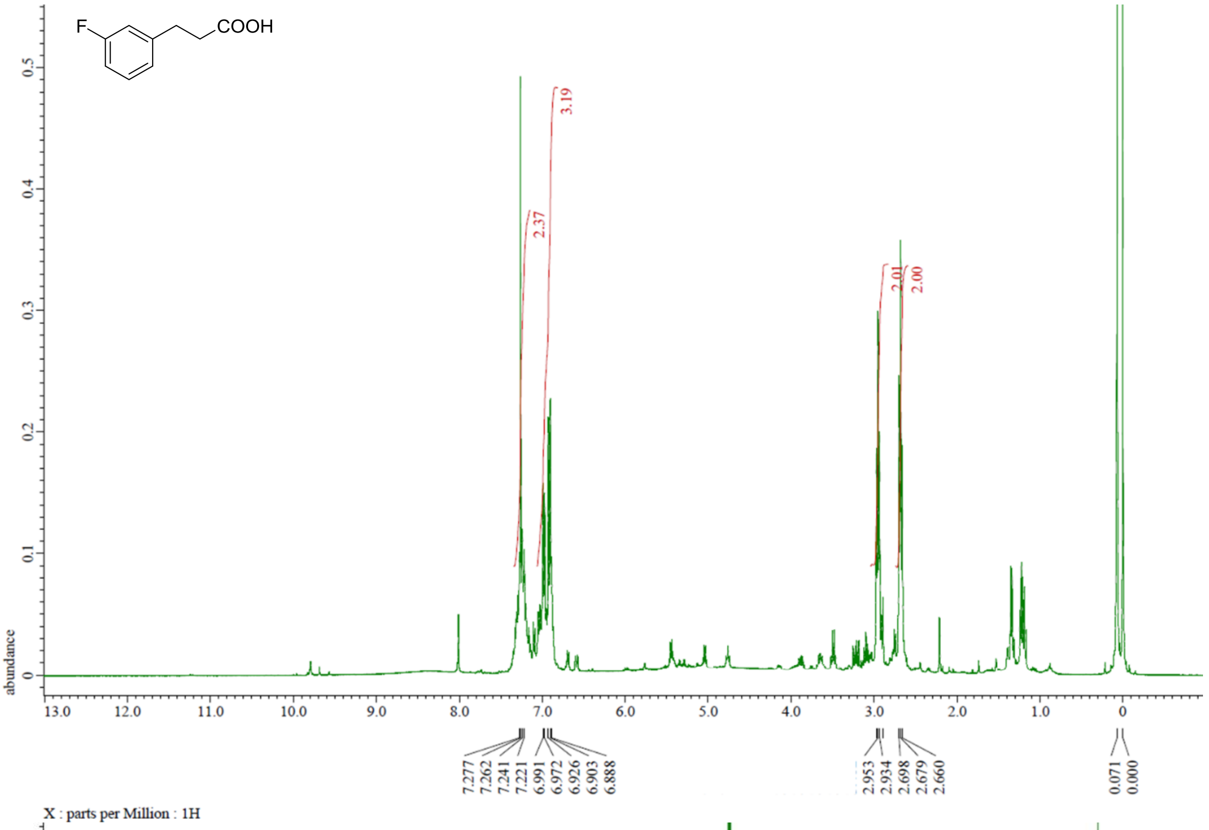
F
COOH
abundance
X : parts per Million : 1H
7.277
7.262
7.241
7.221
6.991
6.972
6.926
6.903
6.888
2.953
2.934
2.698
2.679
2.660
0.071
0.000
2.37
3.19
2.01
2.00

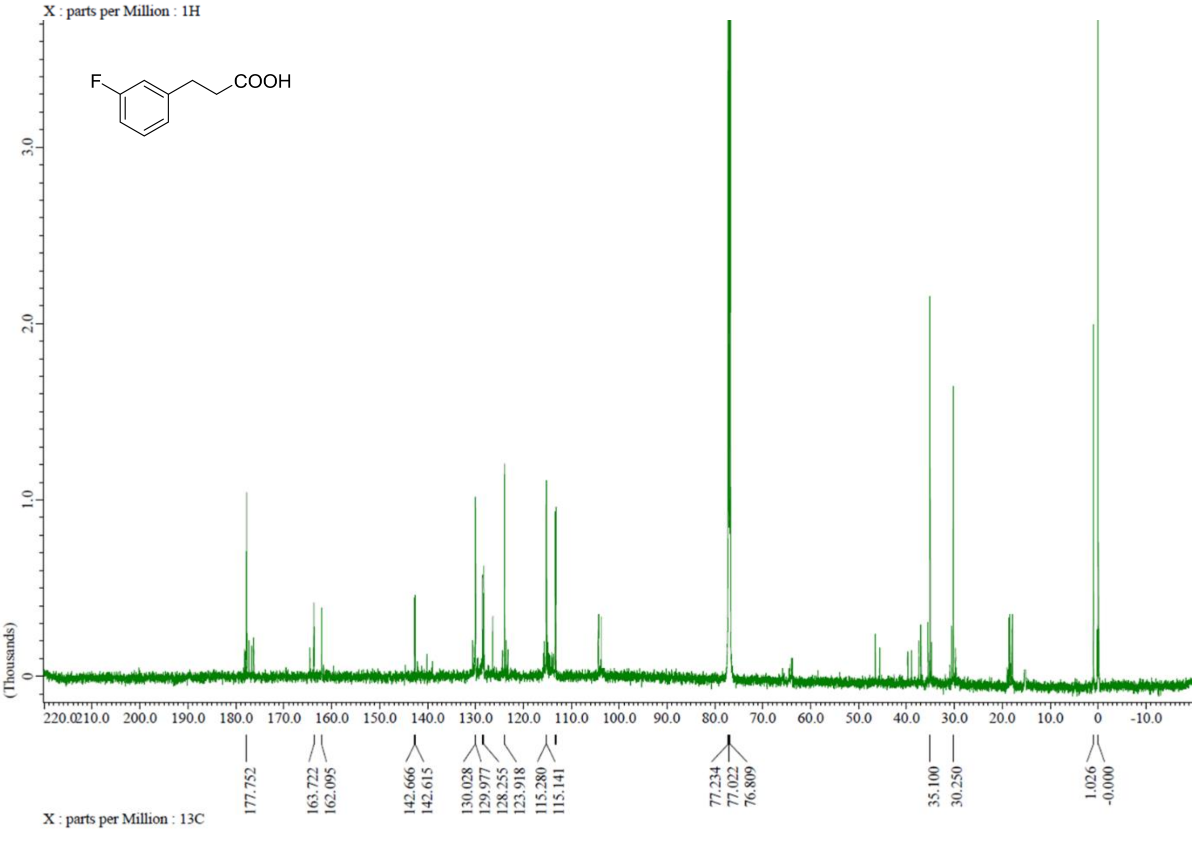
F
COOH
(Thousands)
X : parts per Million : 13C
177.752
163.722
162.095
142.666
142.615
130.028
129.977
128.255
123.918
115.280
115.141
77.234
77.022
76.809
35.100
30.250
1.026
-0.000

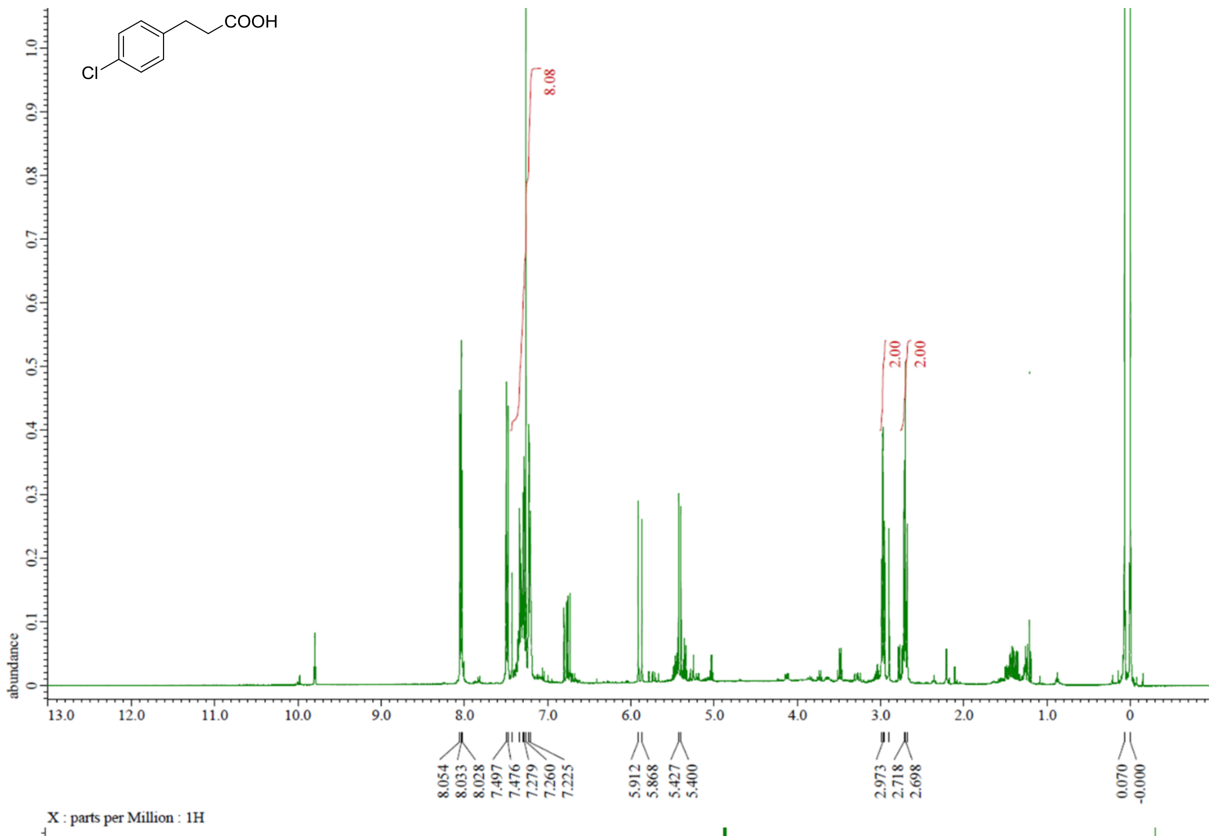
COOH
Cl
8.08
2.00
2.00
abundance
8.054
8.033
8.028
7.497
7.476
7.279
7.260
7.225
5.912
5.868
5.427
5.400
2.973
2.718
2.698
0.070
-0.000
X : parts per Million : 1H

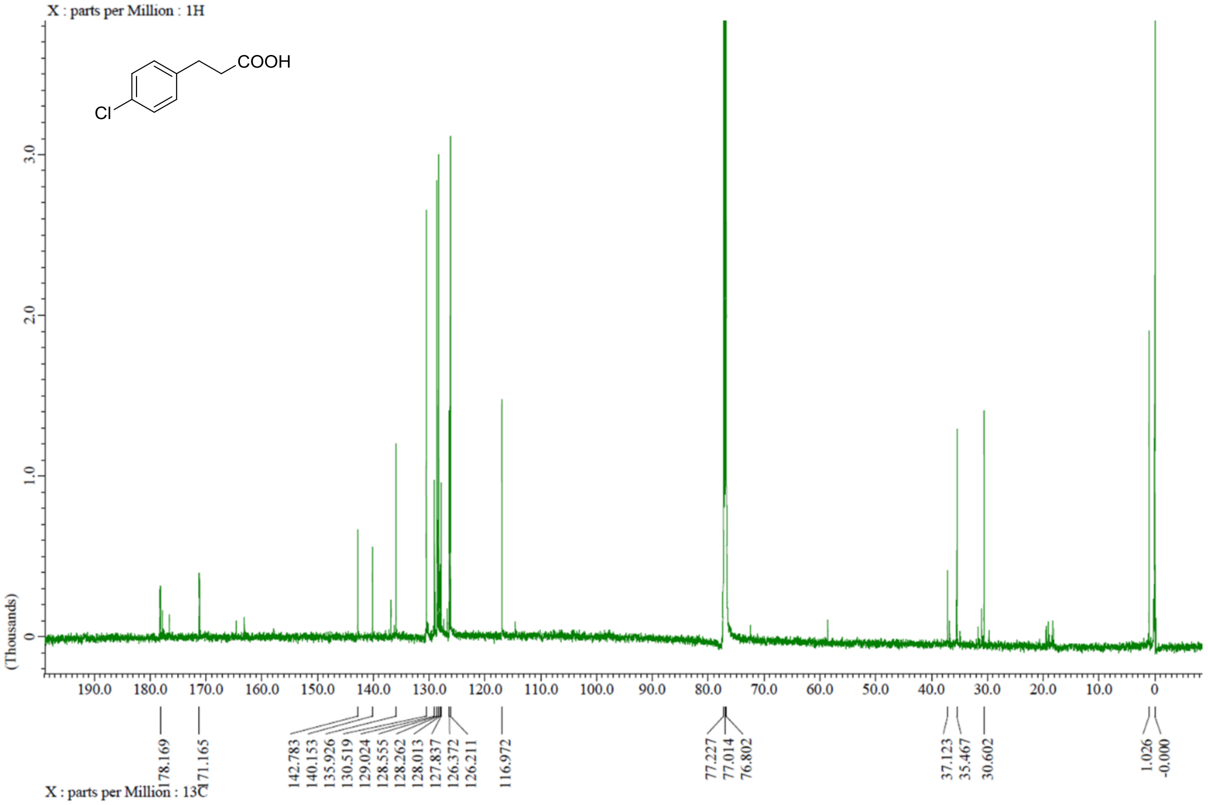
COOH
Cl
(Thousands)
178.169
171.165
142.783
140.153
135.926
130.519
129.024
128.555
128.262
128.013
127.837
126.372
126.211
116.972
77.227
77.014
76.802
37.123
35.467
30.602
1.026
-0.000
X : parts per Million : 13C

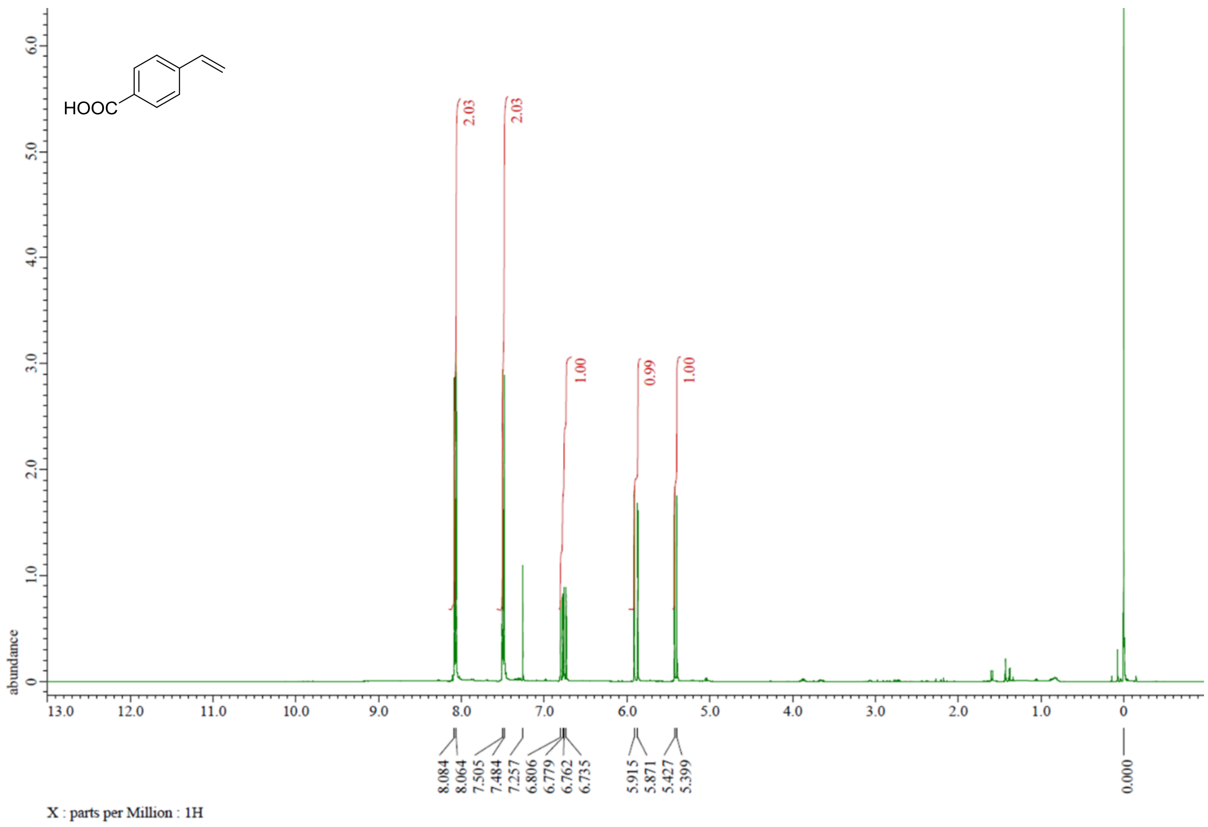
HOOC
abundance
2.03
2.03
1.00
0.99
1.00
8.084
8.064
7.505
7.484
7.257
6.806
6.779
6.762
6.735
5.915
5.871
5.427
5.399
0.000
X : parts per Million : 1H

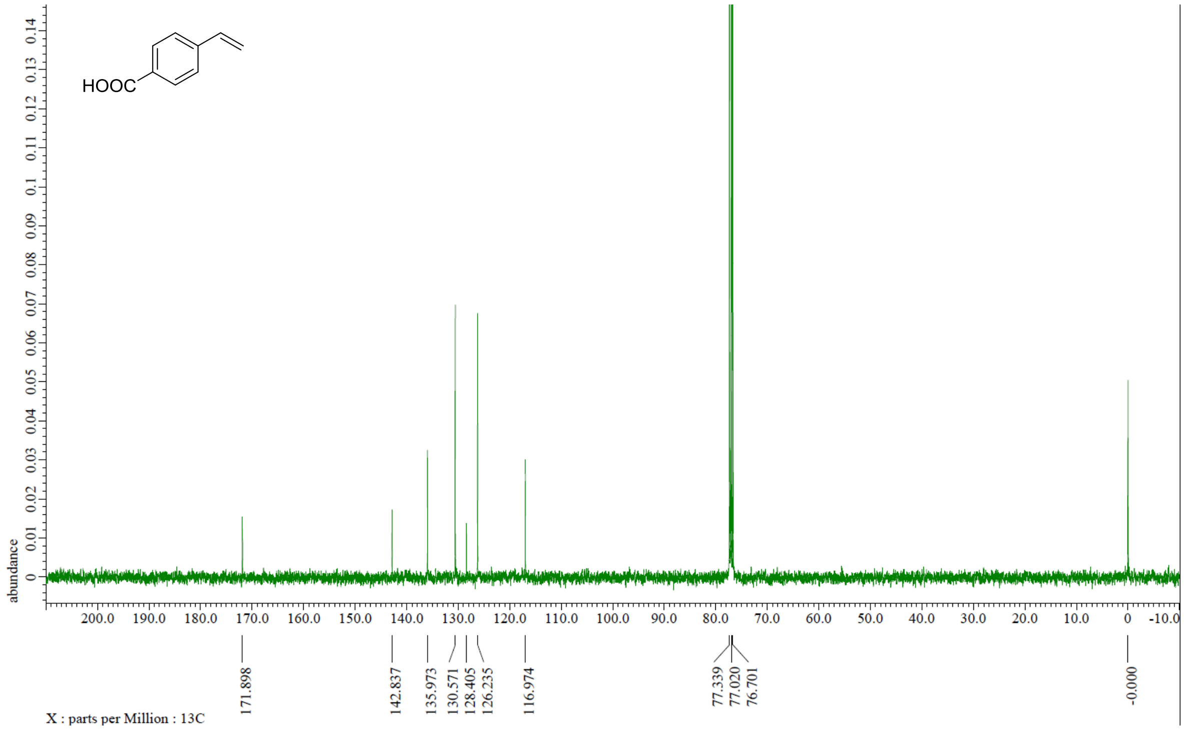
HOOC
abundance
171.898
142.837
135.973
130.571
128.405
126.235
116.974
77.339
77.020
76.701
-0.000
X : parts per Million : 13C

6. Supporting figures and tables

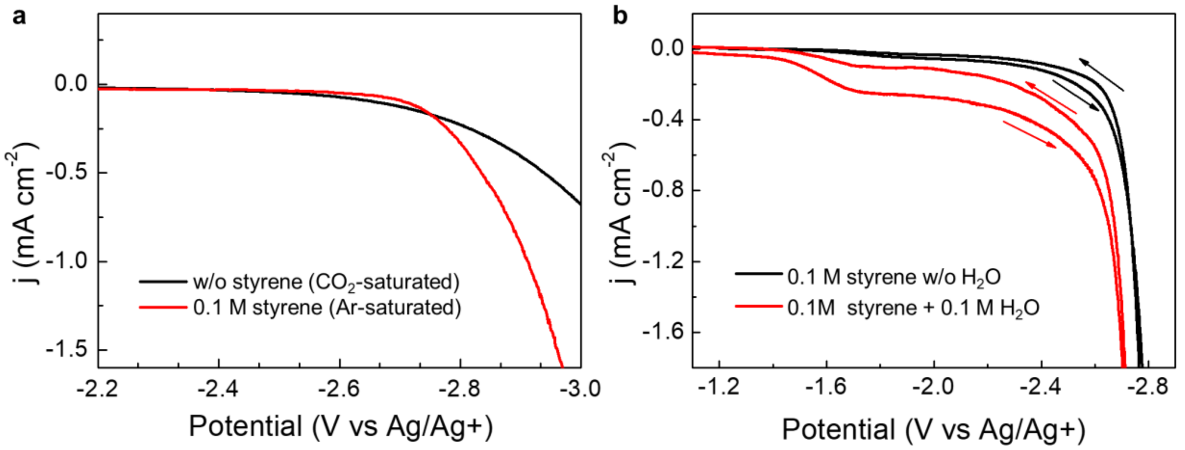

Figure S1. Voltammetric measurements of styrene. The measurements were conducted on a Ni electrode in *N*,*N*-dimethylformamide and tetrabutylammonium tetrafluoroborate (0.1 M) electrolyte at room temperature. **a**, Cathodic linear sweep voltammetry data of CO_2-saturated electrolyte under 1 atm CO_2 and 0.1 M styrene in Ar-saturated electrolyte under 1 atm Ar at 20 mV s^{-1}. **b**, Cyclic voltammetry data of 0.1 M styrene without and with 0.1 M H_2O in CO_2-saturated electrolyte under 1 atm CO_2 at 20 mV s^{-1}.

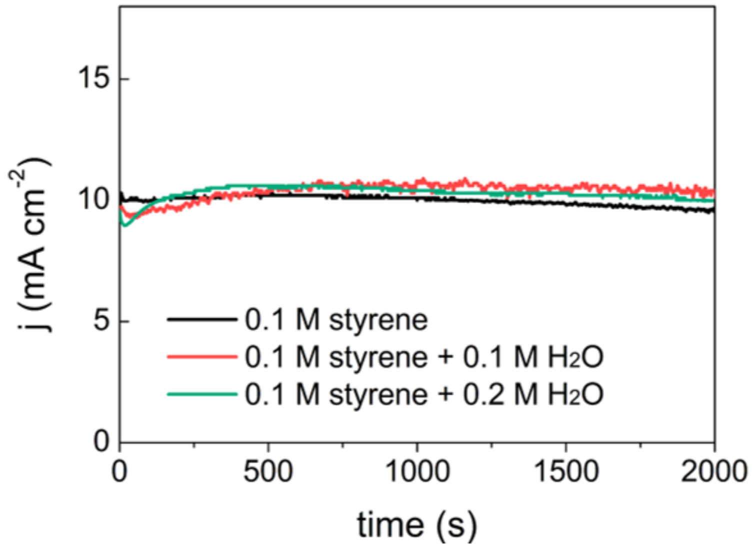

Figure S2. Bulk electrolysis of styrene (0.1 M) with different amounts of water. The electrolysis was conducted on the Ni electrode in CO_2-saturated DMF and $TBABF_4$ (0.1 M) electrolyte at room temperature and under 1 atm CO_2. The current density was 10 mA cm^{-2}, and the total charge of 20 C was passed for approximately 2000 s.

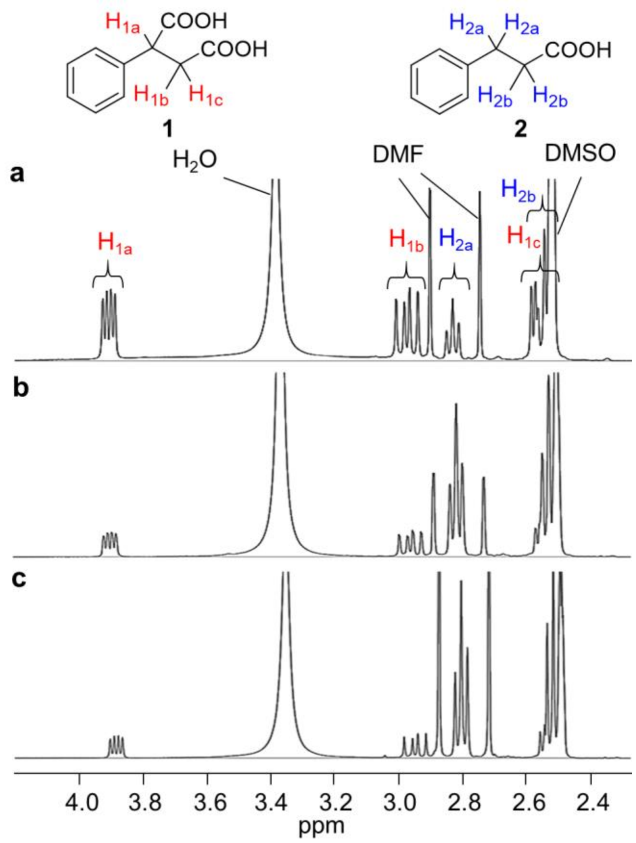

Figure S3. ^{1}H NMR spectra of acid products 1 and 2 from the electrolyte after electrochemical carboxylation (400 MHz, DMSO-*d*6). Peaks depicted as H_{1a}, H_{1b} and H_{1c} refer to protons of **1** and H_{2a} and H_{2b} refer to protons of **2**. Residual solvent peaks from water, DMF and DMSO-*d*6 were also detected. The electrochemical carboxylation of styrene (0.1 M) was conducted on Ni electrode in CO_2 saturated DMF and $TBABF_4$ (0.1 M) electrolyte with j = 10 mA cm^{-2}, charge passed = 20 C, room temperature and 1 atm CO_2 in the presence of **a**, 0.025 M **b**, 0.05 M and **c**, 0.1 M H_2O. The products in the electrolyte were acidified with HCl (2 M) and extracted with ether for ^{1}H NMR analysis.

Table S1. Product yields from the electrochemical carboxylation of styrene [a].

entry	Proton additive (equiv.)	FE of acids (%) [b]		**1 : 2**	FE of gas products (%) [c]		
		**1**	**2**		H_2	CO	CH_4
1	-	89	3.2	97 : 3	0.5	0.8	0.2
2	H_2O (0.25)	55	33	62 : 38	2.6	0.8	1.3
3	H_2O (0.5)	28	59	35 : 65	2.4	0.1	0.6
4	2 M HCl (0.5)	27	61	31 : 69	2.7	0.3	0.4
5	H_2O (1)	26	65	29 : 71	3.2	0.4	0.4
6	2 M HCl (1)	20	68	23 : 77	4.5	0.3	0.6
7	H_2O (2)	15	61	20 : 80	15	0.3	2.0
8	H_2O (3)	9.5	61	14 : 86	17	0.7	6.7
9	H_2O (4)	11	52	17 : 83	30	0.5	3.2
10	H_2O (6)	3.5	58	6 : 94	28	0.6	4.8
11	H_2O (10)	1.8	47	4 : 96	44	0.9	0.3

[a] Reaction conditions : Styrene (0.1 M) in *N*,*N*-dimethylformamide and tetrabutylammonium tetrafluoroborate (0.1 M), Ni cathode, Mg anode, j = 10 mA cm^{-2}, charge passed = 20 C, room temperature and P_{CO2} = 1 atm.

[b] Yields were determined by ^1H NMR after acidification with HCl (2 M) and ether extraction.

[c] Yields were determined by gas chromatography after electrolysis.

FE, Faradaic efficiency; **1**, 2-phenylsuccinic acid; **2**, hydrocinnamic acid.

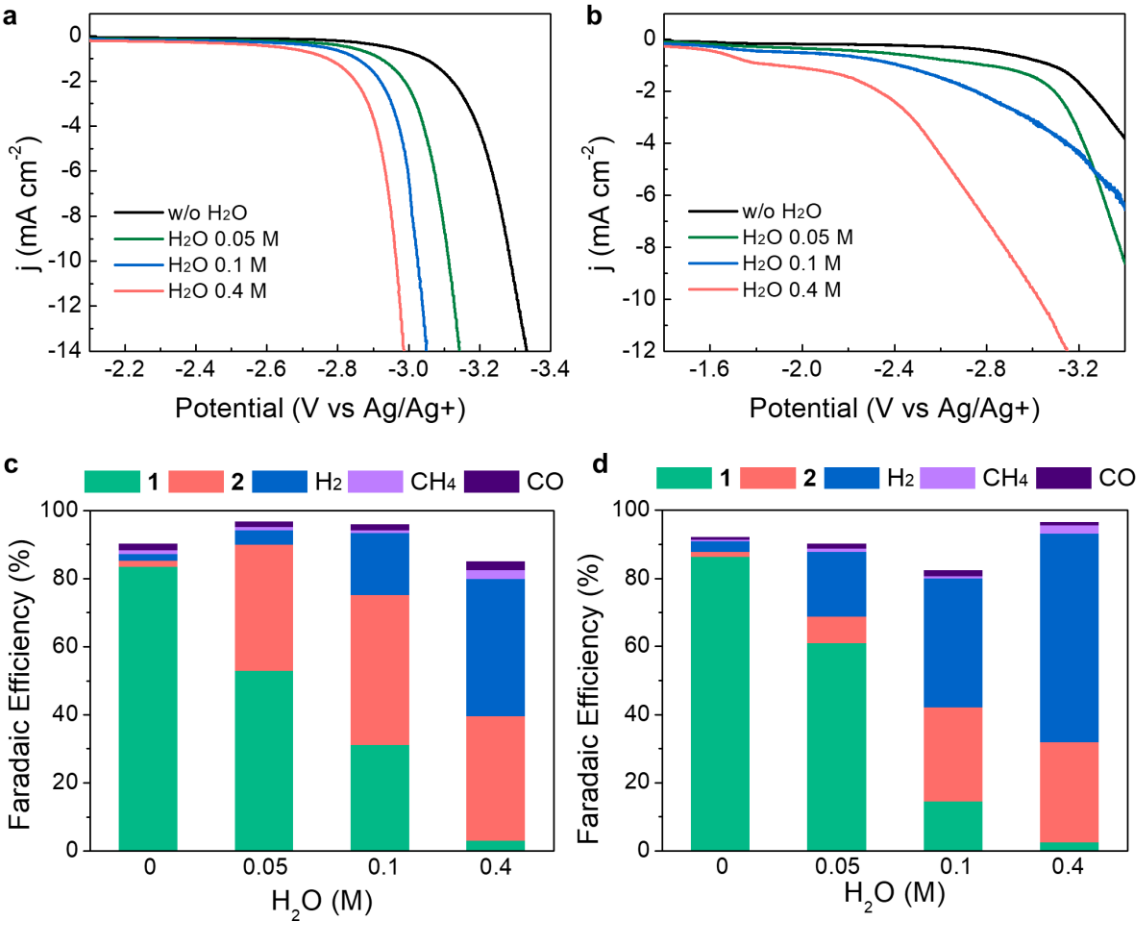

Figure S4. Effect of cathode electrodes. The electrochemical measurements and electrolysis were conducted in CO_2-saturated *N*,*N*-dimethylformamide and tetrabutylammonium tetrafluoroborate (0.1 M) electrolyte with various concentrations of H_2O (0 to 0.4 M) at room temperature and 1 atm CO_2. Cathodic linear sweep voltammetry data of 0.1 M styrene at 20 mV s^{-1} on **a**, Ti electrode and **b**, Pt electrode. Faradaic efficiency (%) of products from electrochemical carboxylation of 0.1 M styrene on **c**, Ti electrode and **d**, Pt electrode. Electrolysis was conducted with j = 10 mA cm^{-2} and charge passed = 20 C. The products in the electrolyte were acidified with HCl (2 M) and extracted with ether for ^1H NMR detection (**1** and **2**). The gas products (H_2, CO and CH_4) in the reactor headspace were detected by gas chromatography.

Table S2. Influence of current density on electrochemical carboxylation of styrene.[a)]

entry	j (mA cm^{-2})	η (mV)	TOF[b)] (s^{-1})	FE of acids (%)[c)]		**1 : 2**	FE of gas products (%)[d)]		
				**1**	**2**		H_2	CO	CH_4
1	2	200	0.018	0.3	0.6	31 : 69	37	1.4	0.5
2	5	270	0.85	9	11	46 : 54	36	1	0.3
3	10	320	10	26	65	29 : 71	3	0.4	0.1
4	35	550	34	22	63	26 : 74	5	1.2	0.3
5	50	760	38	19	50	28 : 72	3	0.6	0.2

[a)] Reaction conditions : Styrene (0.1 M) with H_2O (0.1 M) in *N*,*N*-dimethylformamide and tetrabutylammonium tetrafluoroborate (0.1 M), Ni cathode, Mg anode, charge passed = 20 C, room temperature and P_{CO2} = 1 atm.

[b)] TOF of β-hydrocarboxylation based on the FE of **2**

[c)] Yields were determined by ^{1}H NMR after acidification with HCl (2 M) and ether extraction.

[d)] Yields were determined by gas chromatography after electrolysis.

TOF, turnover frequency; FE, faradaic efficiency; **1**, 2-phenylsuccinic acid; **2**, hydrocinnamic acid.

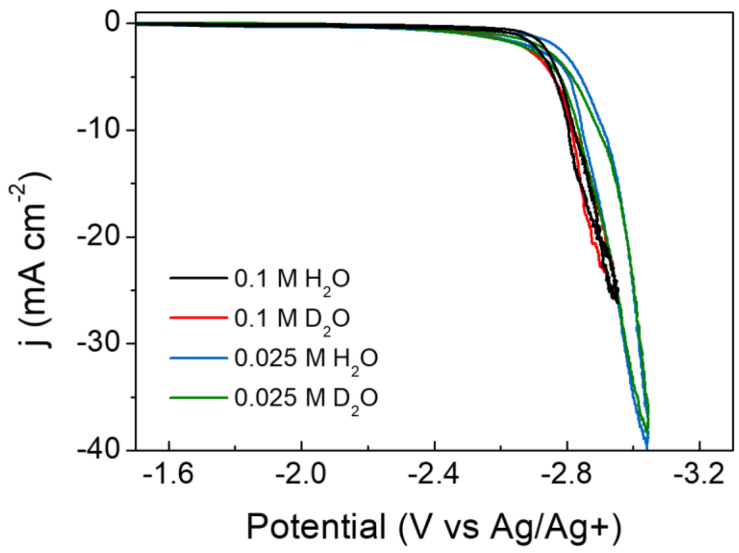

Figure S5. Cyclic voltammetry scans of styrene (0.1 M) at 0.1 V s^{-1} with H_2O (0.1 M) / D_2O (0.1 M) / H_2O (0.025 M) / D_2O (0.025 M). The electrolysis was conducted on a Ni electrode in CO_2-saturated DMF and $TBABF_4$ (0.1 M) electrolyte at room temperature and under 1 atm CO_2.

Figure S6. Influence of radical inhibitors on electrochemical carboxylation of styrene.[a)]

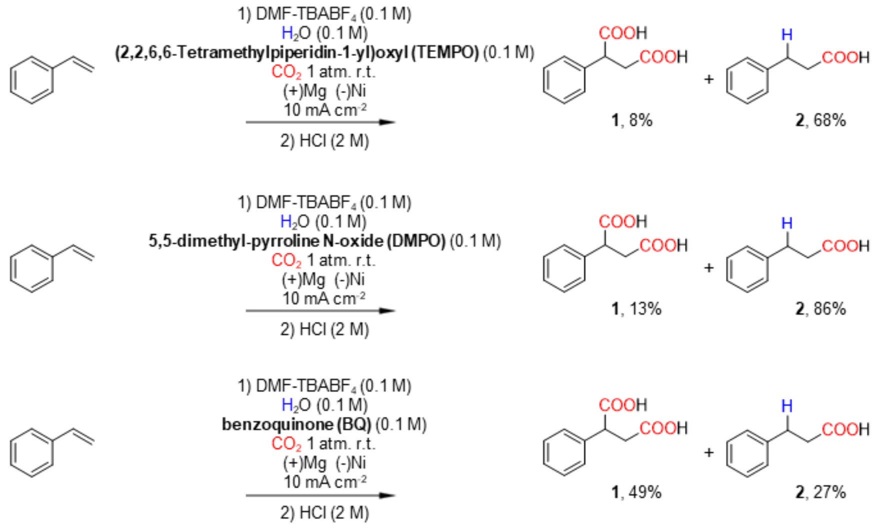

[a)] Reaction conditions : Styrene (0.1 M) with H_2O (0.1 M) in *N*,*N*-dimethylformamide and tetrabutylammonium tetrafluoroborate (0.1 M), Ni cathode, Mg anode, charge passed = 20 C, room temperature and P_{CO2} = 1 atm. Yields were determined by gas chromatography after electrolysis based on the applied charge.

1, 2-phenylsuccinic acid; **2**, hydrocinnamic acid.

7. References

[1] a) F. Song, X. Hu, *J. Am. Chem. Soc.* **2014**, *136*, 16481; b) B. S. Yeo, A. T. Bell, *J. Am. Chem. Soc.* **2011**, *133*, 5587.

[2] J. F. Bell, *Phil. Mag.* **1965**, *11***,** 1135.
